# A Scoping Review of Human Teratogens and Their Impact on the Developing Brain: A Contribution From the ConcePTION Project

**DOI:** 10.1002/bdr2.2497

**Published:** 2025-09-17

**Authors:** M. Bluett‐Duncan, J. Adams, M. Berkovitch, M. Berlin, A. Cahoon, J. Clayton‐Smith, C. Jackson, S. Khanom, D. Mølgaard‐Nielsen, J. L. Richardson, V. Simms, M. Stellfeld, U. Winterfeld, L. M. Yates, R. L. Bromley

**Affiliations:** ^1^ Division of Neuroscience, School of Biological Sciences The University of Manchester Manchester UK; ^2^ Department of Psychology University of Massachusetts Boston Boston MA USA; ^3^ Clinical Pharmacology and Toxicology Unit, TIS Zerifin, Shamir Medical Center (Assaf Harofeh), Zerifin, Affiliated to the School of Medicine, Faculty of Medical and Health Sciences Tel Aviv University Tel Aviv Israel; ^4^ School of Psychology, Faculty of Life and Health Sciences Ulster University Coleraine UK; ^5^ Manchester Centre for Genomic Medicine University of Manchester, St Mary's Hospital Manchester UK; ^6^ The Walton Centre for Neurology and Neurosurgery Liverpool UK; ^7^ Royal Manchester Children's Hospital Manchester University Hospitals NHS Foundation Trust Manchester UK; ^8^ Global Patient Safety, Novo Nordisk A/S Soeborg Denmark; ^9^ UK Teratology Information Service, Newcastle Upon Tyne Hospitals NHS Foundation Trust Newcastle upon Tyne UK; ^10^ Swiss Teratogen Information Service, Clinical Pharmacology Service Lausanne University Hospital and University of Lausanne Lausanne Switzerland; ^11^ The Newcastle Upon Tyne Hospitals NHS Foundation Trust Newcastle upon Tyne UK; ^12^ KRISP, University of KwaZulu‐Natal Durban South Africa; ^13^ Epilepsy Research Institute UK London UK

**Keywords:** neurobehavior, neurodevelopment, pharmacovigilance, pregnancy, teratogen

## Abstract

Certain medications, when used during pregnancy, are known to impact human prenatal development. Historically, little attention has been given to the impact of in utero exposure on the developing brain, despite the significance of known teratogen‐induced neurodevelopmental difficulties. This scoping review systematically identified and extracted neurodevelopmental outcome data for medications with established physical teratogenic effects and synthesized the key study characteristics. Medications with evidence of physical teratogenicity (*n* = 24) were defined by a panel of experts. Eligible studies reporting any neurodevelopmental outcomes following pregnancy exposure to the defined list of human structural teratogens were identified through electronic searches of MEDLINE and EMBASE. We identified 207 studies (254 publications) for inclusion, comprising 81 empirical cohorts and 126 case series. Concerningly, only 13 of 24 (54%) confirmed structural teratogens have been subject to any empirical investigation of neurodevelopmental outcomes. The mean time between authorization of known structural teratogens and the first empirical study investigating neurodevelopmental outcomes using a comparison group and formal data analysis is 33 years (Range: 11–64 years). When neurodevelopmental outcomes are investigated for medication exposures with physical teratogenic signatures, there are high levels of neurodevelopmental alterations (77%). These findings do not speak to a pharmacovigilance system that is functioning efficiently to identify and ameliorate neurodevelopmental risk, even for the medications with identified structural teratogenic risk. Given the high proportion of known physical teratogens exhibiting additional altered neurodevelopmental outcomes and the substantial lifetime burden of such alterations, to the individual and society, the timelines remain too long.

## 1 Background

Certain medications, chemicals and maternal diseases can disrupt processes of human fetal development. Disruption of normal embryonic/fetal developmental trajectories leads to a continuum of outcomes, from those immediately evident, such as embryo loss or major structural malformation, through to minor or internal physical anomalies (Vorhees 1986). Less frequently considered and historically neglected, are the possible effects of in utero exposures on the development of the brain. While disruptions of brain structure including cortical abnormalities, may be detected prenatally or at birth through advances in imaging, functional impairment with no associated visible changes may only become apparent much later on when more complex brain functions have emerged (Vorhees 1986; Adams et al. 2000; Rice and Barone Jr. 2000). Alterations to the development of the neuronal architecture can lead to a myriad of childhood developmental difficulties, including delay in early language and motor skill acquisition, lower IQ, poorer educational outcomes, and an increased rate of clinical diagnoses such as autism spectrum disorder (ASD), attention deficit hyperactivity disorder (ADHD), and intellectual disability, among others (Adams et al. 2000; Rice and Barone Jr. 2000; Bluett‐Duncan et al. 2023). Depending on the unique characteristics of a teratogen (such as half‐life, mode of action), as well as the gestational timing of the exposure, the dose and duration of exposure, together with variable genetic susceptibility factors (Vorhees 1986), the neurodevelopmental or neuropsychological impact can range from mild through to substantial and life impacting.

Alcohol, a long‐established human teratogen, is capable of inducing a wide range of adverse effects on the developing brain (Mattson et al. 2019). Consequently, this places the infant at risk of early developmental delay and later neuropsychological difficulties, such as issues with attention and working memory. In addition to the well‐described wide range of structural malformations (Bromley, Adab, et al. 2023) and dysmorphic facial characteristics, fetal exposure to medicines, such as valproate, phenytoin or isotretinoin can also produce substantial intellectual deficits, social impairments and motor skill disruptions, which vary by the exposure type (Adams and Lammer, 1993; Bromley et al. 2019; Dean et al. 2002).

Despite the significance of these teratogen‐induced neurodevelopmental difficulties, for many exposures there is a mismatch between knowledge of physical teratogenicity and the potential for neurodevelopmental impact. This is largely because many physical abnormalities are present at birth and therefore readily observed and documented, whereas the identification of neurodevelopmental outcomes requires prolonged, targeted follow‐up at key developmental stages (Bromley, Bickle Graz, et al. 2023). Historically, the physical effects of human teratogens have taken extensive periods, in some cases decades, to determine (Adam et al. 2011). This is in part due to the complexity of gathering data over prolonged periods where use of a medication is infrequent and the features subtle or non‐pathognomonic. Identification of neurodevelopmental impact is even more challenging given that neurodevelopmental impairments can be present in the absence of dysmorphic features or structural anomalies, and typically require longer‐term follow‐up to ensure reliable diagnosis. Previously a secondary consideration to the physical outcomes observable at birth, the call to consider the potential impact of teratogens on brain development, even in the absence of physical symptoms, is gathering pace (Bromley, Bickle Graz, et al. 2023).

Despite increasing recognition regarding the need to extend pharmacovigilance in pregnancy to identify causal associations between in utero medication exposure and childhood neurodevelopmental impairments (European Medicines Agency 2019; U.S. Food and Drug Administration 2019), there has previously been limited guidance regarding the methodological approach to do this effectively. An expert working group (EWG) was convened to discuss optimal approaches to measuring neurodevelopmental outcomes in pregnancy pharmacovigilance studies and produced 11 recommendations (Bromley, Bickle Graz, et al. 2023). These recommendations focus on how and when medications should be prioritized for neurodevelopmental follow‐up, and different components of an optimal methodological approach in this area, including the core neurodevelopmental outcomes that should be evaluated before a conclusion can be made. While these recommendations are the first published guidance regarding the collection and analysis of neurodevelopmental outcome data in pregnancy pharmacovigilance, they are aligned with previous consensus initiatives in the domains of childhood vaccines and chemical exposures during pregnancy (Amler et al. 2006; Villagomez et al. 2019).

To further address this evidence gap, this scoping review aimed to systematically identify and extract neurodevelopmental outcome data for medications with established physical teratogenic effects, and to review the key characteristics of the studies investigating these outcomes. Given that brain development is typically impacted at a lower dose than physical development, medications with established physical teratogenicity should have been prioritized for neurodevelopmental follow‐up (Vorhees 1986). The state of the literature regarding these medications will therefore act as an excellent indicator as to the effectiveness of pregnancy pharmacovigilance more generally.

Due to the known physical and neurodevelopmental effects of certain anti‐seizure medications (ASMs) (e.g., valproate) (Bromley et al. 2014; Clayton‐Smith et al. 2019), which led to the establishment of a network of pregnancy registers that have provided access to cohorts of pregnant women and their children (Knight et al. 2023; Thomas, Salim et al. 2022), it is expected that the majority of publications will focus on anti‐seizure medications (ASMs). As a result, and as this is a scoping review, data will be reviewed at a high level across two predefined drug categories, ASMs and non‐ASMs. Where available, publications are reviewed to provide an understanding of the diversity, pattern, and scale of the impact on human brain development. Finally, where appropriate, studies included in this review are evaluated against the recommendations provided by the recent EWG (Bromley, Bickle Graz, et al. 2023) to understand the current state of the literature relative to the proposed optimal criteria for pregnancy pharmacovigilance.

## 2 Methods

### 2.1 Protocol

The protocol for the review was drafted by the authors and the final version was registered with the Open Science Framework on May 25, 2022. The protocol is available from https://osf.io/9rfb4/.

### 2.2 Inclusion Criteria

#### 2.2.1 Publication Types

The data from original research articles, case reports, case series and letters with original data on one or more children were included. Conference abstracts were outside the scope of this review.

#### 2.2.2 Study Design

This review included studies with at least one exposed individual, including case studies, where at least one aspect of neurodevelopment is reported upon.

The following study designs were included:

1. Prospective observational cohort studies.
2. Retrospective observational cohort studies.
3. Case control studies.
4. Randomized controlled trials.
5. Case reports or case series.

This review permitted the inclusion of studies which utilized data collected for a bespoke analysis of medication safety in pregnancy (primary data) and for data which were originally collected for alternative reasons, such as population administrative purposes (e.g., health or education; secondary data).

Case series/studies that reported outcomes for patients exposed to either duo‐ or polytherapy treatments are extremely common and were included as these publications serve an important role in generating risk signals in pregnancy pharmacovigilance. By including only case reports/series reporting monotherapy exposures, we would be excluding key pieces of evidence and would not be able to provide a comprehensive review of how neurodevelopmental evidence has evolved.

#### 2.2.3 Exposures

Medications with evidence of ability to disrupt the physical development of the human fetus, either through the inducement of structural congenital malformations, facial dysmorphism or alterations in fetal growth were determined by a panel of experts within the European Network of Teratology Information Services' (ENTIS) (Table 1). Consensus was required from at least three ENTIS member sites for inclusion in this scoping review.

**TABLE 1:** Structural teratogens included in this review and the number of case series/reports and empirical cohorts identified.

Medication	Physical effects	Case series (*n*)	Empirical cohorts (*n*)
**Anti‐Seizure**			
Carbamazepine	Variable by individual medication type but include cardiovascular (phenobarbital, primidone, valproate), neural tube (valproate, carbamazepine), skeletal (valproate), orofacial cleft (topiramate, valproate) and limb (valproate). Facial dysmorphia (phenytoin, carbamazepine, valproate). Growth disruption (topiramate).	15	42
Fosphenytoin		0	0
Phenytoin		32	24
Phenobarbital		20	22
Primidone		9	5
Topiramate		0	16
Valproate		40	44
**Anticoagulant**			
Acenocoumarol	Multiple malformations including nasal hypoplasia, stippled epiphyses, skeletal and digital. Growth disruption. Facial dysmorphia.	2	0
Phenindione		0	0
Warfarin		15	2
**Antithyroid**			
Carbimazole	Multiple malformations including skin defects including aplasia cutis, choanal atresia, esophageal atresia, other malformations of the gastrointestinal tract. Facial dysmorphia.	3	1
Methimazole		11	2
**Immunosuppressive**			
Methotrexate and Aminopterin	Multiple malformations including skeletal, cardiovascular, urogenital, holoprosencephaly. Growth disruption.	7	0
Mycophenolate	Multiple malformations including orofacial cleft, microtia, external auditory canal atresia, micrognathia, cardiovascular, esophageal atresia.	5	0
**Oral Retinoids**			
Acitretin	Multiple malformations including central nervous system abnormalities, orofacial clefts, cardiovascular, skeletal, limb and ear. Facial dysmorphia.	1	0
Alitretinoin		0	0
Bexarotene		0	0
Isotretinoin		8	2
Tretinoin		0	0
**Other Medications**			
Diethylstilbesterol	Malformations of the female and male reproductive organs.	0	7
Misoprostol	Moebius syndrome. Multiple malformations including cranial bone defects, ophalocele, and gastroschisis.	4	2
Thalidomide	Thalidomide: multiple malformations including limb, cardiovascular, ocular and microtia. Risk is less clear for the analogs.	2	6
Lenalidomide		0	0

#### 2.2.4 Participants

Studies, which report on children, adolescents, or adults who were exposed in utero, at any gestational time, to the defined list of teratogenic medications (Table 1) were eligible for inclusion.

#### 2.2.5 Outcomes

The term “neurodevelopment” covers a broad range of outcomes, which are measured by an equally broad set of tools or observations. Given that this review seeks to undertake a scoping exercise, no limitations were placed on neurodevelopmental outcome type.

### 2.3 Exclusion Criteria

Studies were excluded where:

- They covered paternal exposures only.
- Empirical studies did not report monotherapy drug specific neurodevelopmental outcomes (e.g., outcomes are reported for a group of medications or in the context of polytherapy exposure).

### 2.4 Search Strategy

Eligible studies were identified through electronic searches of MEDLINE (Ovid) and EMBASE (Ovid) in August 2020. Follow‐up searches were completed in February 2023 and June 2024 to ensure that all relevant articles were included. Results were limited to the English language.

The search terms were developed in line with PICO guidance (Richardson et al. 1995) which led to the creation of 3 levels: Level 1 determined that the exposure was during pregnancy or when the participant was a fetus; Level 2 outlined the relevant exposures, and Level 3 the included outcomes. The comparator group was not defined here due to the scoping nature of this review. The Medical Subject Heading (MeSH) and Key Words of 10 articles known to meet the inclusion criteria were reviewed at http://mesh.med.yale.edu. The search terms can be reviewed at https://osf.io/9rfb4/. The search strategy was first developed for Medline on the OVID platform and then modified for EMBASE. Reprotox, a specialist teratology reference database (The Reprotox System, n.d.), was used to validate the search results and identify additional references. Reference lists of relevant review articles were hand searched to identify additional studies.

### 2.5 Study Selection

The search results were downloaded into Covidence Software (www.covidence.org) and duplicates were removed. Title and abstract screening of each retrieved article was completed independently by two authors (MS, RB, DM‐N, RF, JLR, AC, CJ, and MBD). Articles which met the inclusion criteria were selected for full‐text review. Full‐text review of each article was also completed independently by two authors (MBD, MS, RB, DM‐N, JLR, AC, and CJ). Any disagreements at either stage were discussed and resolved and, where necessary, the opinion of a third author was sought (RB). Different papers arising from the same cohort were considered together, with the earliest publication selected as the key reference.

**FIGURE 1:**
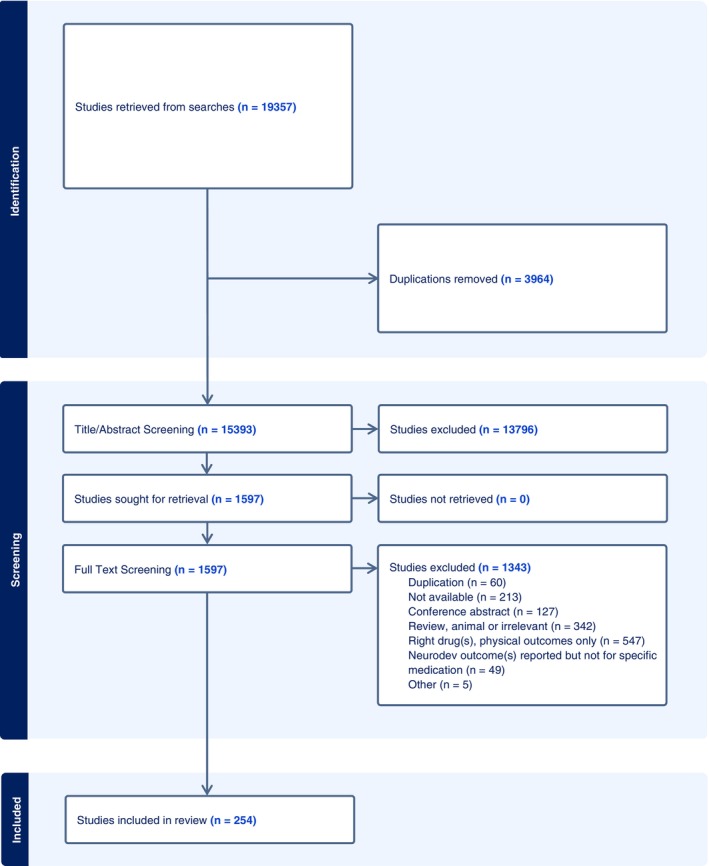
PRISMA flowchart showing the study selection and screening process.

Initial searches identified 19,357 publications (Figure 1). Following the removal of duplicates (*n* = 3964) 15,393 were retained for title/abstract screening, resulting in the removal of a further 13,796 publications. Full text review was carried out for 1597 publications. A total of 1343 publications were excluded at this stage, and 254 publications were deemed eligible for inclusion in this review.

### 2.6 Data Extraction

Data extraction of the eligible studies was undertaken using an extraction datasheet designed specifically for this review and pre‐registered with the protocol.

Two authors (MBD and SK) completed extraction of a sample of included studies and made a note of any issues and any areas/variables that need clarifying or modifying. These were discussed with another member of the research team (RB) to reach consensus on the issues raised. Following this discussion, any required modifications were made to the template.

This final extraction framework was uploaded to Covidence and data from each study was extracted independently by two reviewers (MBD, SK or JLR). The extracted data were reviewed for consensus using the Covidence tool, and any disagreements were resolved through discussion.

Due to the review's scoping nature, potential bias arising from specific aspects of the study design were not taken into account.

### 2.7 Definitions

Altered Outcomes – Any neurodevelopmental outcome reported to be significantly and negatively affected by medication exposure.

Prospective – Any study in which participants were recruited at any point during pregnancy.

Retrospective – Any study in which participants were recruited at any point post‐birth.

Primary Data – Data collected directly from participants, specifically for research purposes.

Secondary Data – Data originally collected for other purposes, such as population administrative purposes (e.g., health or education), and re‐purposed for research.

Blinding – Was the researcher who administered the assessment unaware (blinded) or aware (non‐blinded) of the exposure status of the participant?

Infant Global Developmental Delay – delays in the attainment of developmental milestones, including motor, communication and cognitive skills.

### 2.8 Comparison to Expert Working Group Publication

The availability of evidence for different medications was compared to 11 Expert Consensus recommendations (Bromley, Bickle Graz, et al. 2023) pertaining to aspects of neurodevelopmental investigation. The breadth and level of evidence for each of the included medications was rated against key recommendations to allow for a meaningful comparison regarding the comprehensiveness of available data for each medication type. Key recommendations are that the range of studies that make‐up the evidence‐base should include:

- Assessment of core neurodevelopmental domains, including cognitive, motor, behavior and emotional functioning as well as clinical disorders and educational outcomes.
- Use of direct standardized assessment, by a researcher blinded to exposure status.
- Consideration of the effects of dose and gestational timing of the medication.
- Data from infancy through to adolescence.
- Use of prospective design.
- Comparator groups with different medication exposures for the same maternal disease(s) and groups with no medication exposure and no maternal disease exposure.
- Core confounding variables, as defined by the expert working group, accounted for in analyses.

## 3 Results

A total of 254 publications, documenting 207 unique cohorts, met the review's inclusion criteria. This included data from 81 empirical cohorts reported in 128 publications, and 126 case series describing 380 individuals. Publications from the same cohort are grouped together and described using either the formal cohort name or another key identifier (e.g., NEAD, Danish Cohort, Israeli TIS etc.). Single publication studies are described using the first author name and date. Empirical studies were grouped into Anti‐Seizure Medicines (ASMs) and Other Medications. Case series are not discussed in detail, but a summary of reported outcomes is provided. Table 1 shows the number of identified publications for each structural teratogen identified by the expert group.

### 3.1 Empirical Studies

**FIGURE 2:**
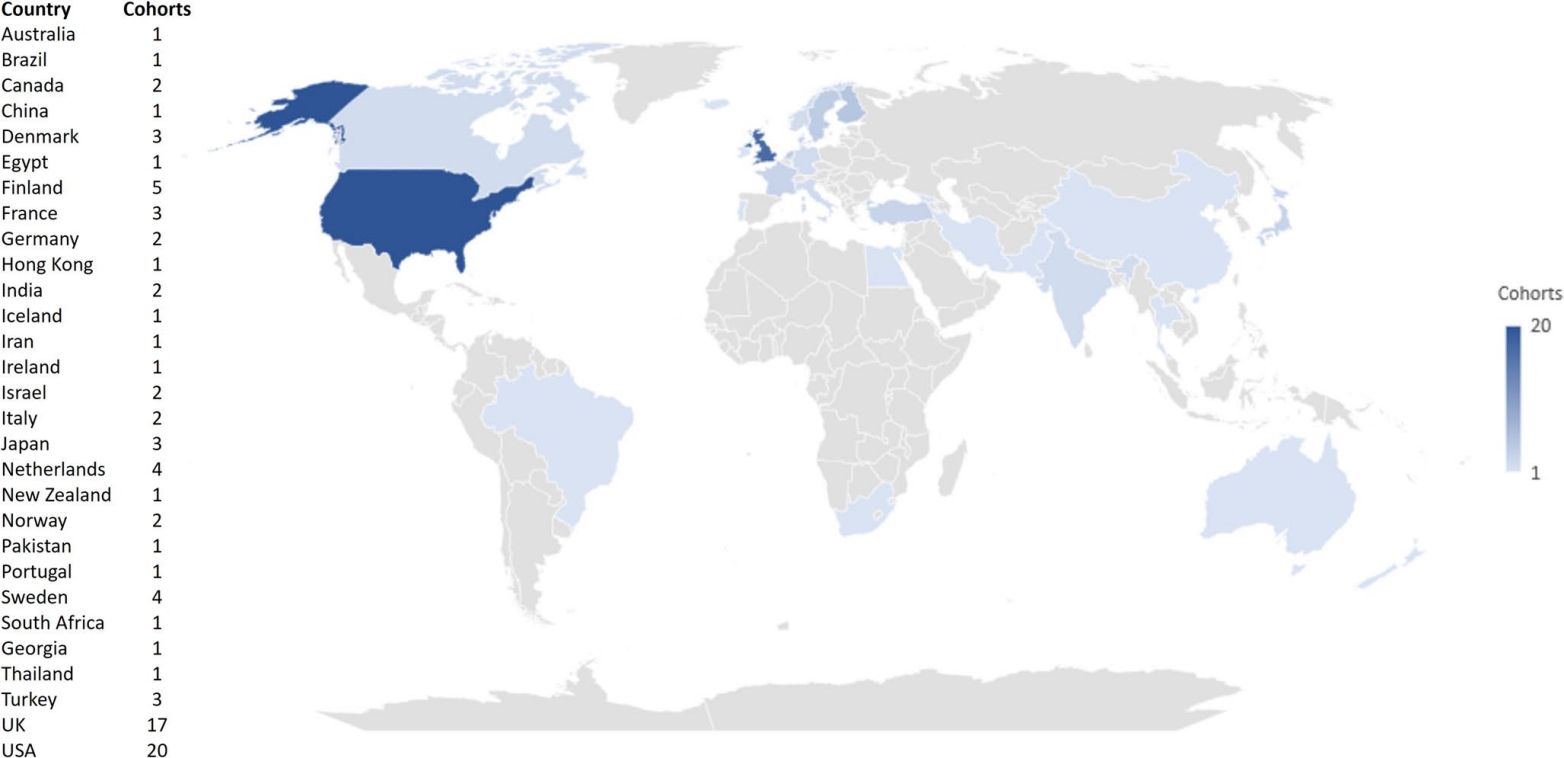
Map showing the location of empirical cohorts included in this scoping review. Note that there were two multinational studies and that in both cases all individual participating countries are shown here.

Eighty‐one empirical cohorts were included in this review. Fifty‐nine (73%) investigated exposure to ASMs (Bluett‐Duncan et al. 2023; Bromley et al. 2019; Thomas, Salim et al. 2022; Adab et al. 2001; Adab et al. 2004; Adams et al. 2022; Asranna et al. 2018; Baker et al. 2015; Barton et al. 2018; Bech et al. 2018; Bjork et al. 2018; Bjork et al. 2022; Blotiere et al. 2020; Bromley et al. 2016; Bromley et al. 2008; Bromley et al. 2010; Bromley et al. 2013; Christensen et al. 2013; Christensen et al. 2019; Cohen et al. 2011; Cohen et al. 2013; Cohen et al. 2019; Coste et al. 2020; Cummings et al. 2011; Daugaard et al. 2020; Deshmukh et al. 2016; Elkjaer et al. 2018; Eriksson et al. 2005; Gaily et al. 2004; Gopinath et al. 2015; Honybun et al. 2021; Huber‐Mollema et al. 2019; Huber‐Mollema et al. 2020; Husebye et al. 2018; Husebye et al. 2020; Kantola‐Sorsa et al. 2007; Kini et al. 2006; Koch et al. 1996; Koch et al. 1999; McVearry et al. 2009; Meador et al. 2010; Meador et al. 2012; Meador et al. 2013; Meador et al. 2011; Nadebaum et al. 2011a; Nadebaum et al. 2011b; Ornoy and Cohen 1996; Ren et al. 2022; Rihtman et al. 2012; Rihtman et al. 2013; Shallcross et al. 2014; Shallcross et al. 2011; Thomas et al. 2008; Thomas, Jeemon et al. 2022; Thomas et al. 2007; Unnikrishnan et al. 2020; Veiby, Daltveit et al. 2013; Veiby, Engelsen, et al. 2013; Viinikainen et al. 2006; Vinten et al. 2005; Vinten et al. 2009; Wide et al. 2002; Wide et al. 2000; Wood et al. 2015; Arkilo et al. 2015; Arulmozhi et al. 2006; Burger et al. 2022; Chainirun et al. 2021; Charlton et al. 2017; Dean et al. 2002; Dessens et al. 1998; Dessens et al. 2000; Forsberg et al. 2011; Gaily et al. 1988; Guveli et al. 2015; Hill et al. 1974; Jones et al. 1989; Kasradze et al. 2017; Kelly et al. 1984; Kishk et al. 2019; Lacey et al. 2018; Lajeunie et al. 2001; Meador et al. 2021; Millar and Nevin 1973; Mohd Yunos and Green 2018; Moore et al. 2000; Parisi et al. 2003; Putignano et al. 2019; Rasalam et al. 2005; Reinisch et al. 1995; Richards et al. 2019; Scolnik et al. 1994; Shankaran et al. 2002; Shankaran et al. 1996; Shapiro et al. 1976; Thorp et al. 2003; van der Pol et al. 1991; Videman et al. 2016; Wiggs et al. 2020; Yigin et al. 2021; Meador et al. 2009; Yasguclukal et al. 2023; Dreier et al. 2023; Hernández‐Díaz et al. 2024; Li et al. 2023; Soomro et al. 2024), seven (9%) investigated diethylstilbesterol (DES) (Kioumourtzoglou et al. 2018; Lish et al. 1991; Reinisch and Sanders 1992; Soyer‐Gobillard et al. 2016; Vessey et al. 1983; Wilcox et al. 1992; Hines and Sandberg 1996), six (7%) investigated thalidomide (Imai et al. 2014; Imai et al. 2020; Kanno, 1987, McFie and Robertson 1973; Mongeau et al. 1966; Nippert et al. 2002), three (4%) investigated anti‐thyroid medications (Azizi et al. 2002; Eisenstein et al. 1992; McCarroll et al. 1976), two (2%) investigated anticoagulant medications (Chong et al. 1984; Wong et al. 1993), two (2%) investigated oral retinoids (Adams and Lammer, 1993; Mitchell et al. 1995), and two (2%) investigated misoprostol (Escumalha et al. 2005; Guedes 2014). Most studies were carried out in higher‐income countries, with the USA (*n* = 20) and the UK (*n* = 17) being the most common settings (Figure 2).

#### 3.1.1 Methodological Aspects of Empirical Studies

Key methodological aspects of identified papers are presented in Table 2 (see Supplementary Tables [Link], [Link] for more details). For the included ASMs, results from 17 cohorts were reported in multiple publications (*n* = 64) and results from 42 cohorts were reported in a single publication. There was a substantially smaller dataset for all other medications associated with physical teratogenic signatures in comparison to the ASMs, even for other central nervous system– acting medications. Due to the limited number of included cohorts, methodological components for other medications are reported together. For these medications, all 22 cohorts reported outcomes in a single publication.

**TABLE 2:** Key methodological features of included studies.

Methods	Anti‐seizure medications			Other medications	Overall (*n* = 81)
	Individual cohort studies (*n* = 42)	Grouped cohort studies (*n* = 17)	All studies (*n* = 59)	All studies (*n* = 22)	
Sample size range	11–4,292,539	27–4,494,926	11–4,494,926	22–47,540	11–4,494,926
Age range	7d‐39y	2 m‐19y	7d‐39y	6 m‐54y	7d‐54y
**Study design**					
Prospective	25 (60%)	16 (94%)	41 (69%)	7 (32%)	48 (59%)
Retrospective	16 (38%)	1 (6%)	17 (29%)	15 (68%)	32 (40%)
Prospective and Retrospective	1 (2%)	0 (0%)	1 (2%)	0 (0%)	1 (1%)
**Study setting**					
Community	6 (14%)	9 (53%)	15 (25%)	7 (32%)	22 (27%)
Hospital	29 (69%)	4 (24%)	33 (56%)	15 (68%)	48 (59%)
Population database	7 (17%)	4 (24%)	11 (19%)	0 (0%)	11 (14%)
**Data type**					
Primary	33 (79%)	13 (76%)	46 (78%)	21 (95%)	67 (83%)
Secondary	9 (21%)	3 (18%)	12 (20%)	1 (5%)	13 (16%)
Primary and Secondary	0 (0%)	1 (6%)	1 (2%)	0 (0%)	1 (1%)
**Exposure data**					
Hospital records	26 (62%)	6 (35%)	32 (54%)	16 (73%)	48 (59%)
Maternal report	13 (31%)	10 (59%)	23 (39%)	5 (23%)	28 (35%)
Pharmacy records	2 (5%)	1 (6%)	3 (5%)	0 (0%)	3 (4%)
Not Reported	1 (2%)	0 (0%)	1 (2%)	1 (5%)	2 (2%)
**Outcome data** ^**a**^					
Education system	3 (7%)	3 (18%)	6 (10%)	1 (5%)	7 (9%)
Health professional	15 (36%)	3 (18%)	18 (31%)	3 (14%)	21 (26%)
Researcher blinded	13 (31%)	11 (65%)	24 (41%)	1 (5%)	25 (31%)
Researcher non‐blinded	14 (33%)	5 (29%)	19 (32%)	11 (50%)	30 (37%)
Parent	3 (7%)	7 (41%)	10 (17%)	6 (27%)	16 (20%)
Teacher	0 (0%)	1 (6%)	1 (2%)	4 (18%)	5 (6%)
Self‐Report	0 (0%)	0 (0%)	0 (0%)	4 (18%)	4 (5%)
Not reported	1 (2%)	0 (0%)	1 (2%)	0 (0%)	1 (1%)
**Comparison Group** ^**b**^					
Unexposed, general population	25 (60%)	14 (82%)	39 (66%)	9 (41%)	48 (59%)
Unexposed, disease matched	10 (24%)	7 (41%)	17 (29%)	5 (23%)	22 (27%)
Other Medication Exposed	12 (29%)	12 (71%)	24 (41%)	0 (0%)	24 (30%)
Other	1 (2%)	0 (0%)	1 (2%)	1 (5%)	2 (2%)
None	8 (19%)	0 (0%)	8 (14%)	6 (27%)	14 (17%)
**Any covariates included in final analysis**					
Yes	19 (45%)	13 (76%)	32 (54%)	2 (9%)	34 (42%)
No	23 (55%)	4 (24%)	27 (46%)	20 (91%)	47 (58%)
**Dose investigation reported**					
Yes	8 (19%)	12 (71%)	20 (34%)	1 (5%)	21 (26%)
No	34 (81%)	5 (29%)	39 (66%)	21 (95%)	60 (74%)
**Timing investigation reported**					
Yes	1 (2%)	5 (29%)	6 (10%)	1 (5%)	7 (9%)
No	41 (98%)	12 (71%)	53 (90%)	21 (95%)	74 (91%)

^a^ Some studies included data from multiple sources and so the total is more than the sum of the studies.

^b^ Some studies included multiple comparison groups and so total is more than sum of studies.

There are some similarities in methodological approaches used in investigating ASMs and other medications, but there are also a number of stark differences (Table 2). For example, the majority of ASM studies utilized a prospective design (70%), while most other medication studies were retrospective (68%), and while 41% of ASM studies included at least some outcome data from assessments conducted by a researcher blinded to the child's exposure status, only 5% of other medication studies did the same. It should also be noted, though, that other medication studies made use of a wider array of informants, including parents (27%) teachers (18%) and self‐report (18%), than in ASM exposure studies (parents = 7%, teachers = 2%, self‐report = 0%). Studies conducted across medication types were generally carried out in similar settings, with hospitals being the most common context for investigations for both ASMs (56%) and other medications (68%). However, ASM investigations appear to have made additional use of national population‐based health databases (19%), an approach which did not feature at all for other included medications. The majority of studies in both categories did utilize at least one comparison group (ASM = 86%, Other = 73%), but the inclusion of covariates in final analyses was far more common in ASM studies (54%) than in other studies (9%). Finally, investigations of dose‐dependent associations and the impact of gestational timing were not present in most studies across all medications but were more often seen in ASM studies (dose = 34%, timing = 10%) than in other medication studies (dose = 5%, timing = 5%).

Differences in the approaches between ASM and other medication studies which met eligibility for inclusion in this review are further demonstrated by the comparison in Table 3 which maps the methodological features of studies for each medication type against the expert recommendations (Bromley, Bickle Graz, et al. 2023). Identified studies investigating neurodevelopmental outcomes following exposure to ASMs with documented teratogenic potential met the suggested requirements for optimal investigations far more consistently than do studies for other (non‐ASM) teratogenic medications.

**TABLE 3:** Mapping existing studies for each medication class onto recommendations for optimal follow‐up of neurodevelopmental outcomes in pregnancy pharmacovigilance produced by a recent expert working group (Bromley, Adab, et al. 2023; Bromley, Bickle Graz, et al. 2023).

Criteria	Scoring	ASMs	Anticoagulant	Antithyroid	DES	Misoprostol	Oral Retinoid	Thalidomide
Evidence base to include assessment of core domains	✓ = 1–2 domains ✓✓ = 3–4 domains ✓✓✓ = 5 domains	✓✓✓	✓	✓	✓✓	✓	✓✓	✓✓
Direct, standardized assessment, by researcher blinded to exposure status.	✓ = 1–2 studies ✓✓ = 3+ studies	✓✓	X	X	✓	X	✓	X
Consideration of dose/timing effects	✓ = ≥ 1 study includes Timing OR Dose ✓✓ = ≥ 1 study includes Timing AND Dose	✓✓	X	X	✓	X	X	X
Evidence base should include data from infancy through to adolescence	✓ = ≥ 1 study in infancy OR adolescence ✓✓ = ≥ 1 study in infancy AND adolescence	✓✓	✓✓	✓✓	✓	✓	✓	✓✓
Prospective Design	✓ = 1–2 studies ✓✓ = 3+ studies	✓✓	✓	✓	✓	✓	✓	X
Comparison: Other medication exposed/unexposed, same disease	✓ = 1–2 studies ✓✓ = 3+ studies	✓✓	X	✓	✓✓	✓	X	X
Comparison Group: Non exposed, general population	✓ = 1–2 studies ✓✓ = 3+ studies	✓✓	✓	✓	✓✓	X	✓	✓
Core Confounding Variables	✓ = ≥ 1 variable in 1–2 studies ✓✓ = ≥ 1 variable in 3+ studies	✓✓	X	X	✓	X	X	X

*Note:* Scoring Key: Green = High Adherence, Yellow = Moderate Adherence, Orange = No Adherence. ASM = Anti‐seizure medication; DES = Diethylstilbestrol; All Outcomes (ASMs + Others) – Assessed and Altered.

#### 3.1.2 Neurodevelopmental Outcomes in Empirical Studies

The most commonly reported neurodevelopmental outcomes across all included cohorts (*n* = 81) were IQ/Intellectual Functioning (*n* = 38, 47%) followed by Infant Global Development (*n* = 36, 44%), Language Development (*n* = 25, 31%), Behavior Problems (*n* = 25, 31%), and Fine or Gross Motor Skills (*n* = 20, 25%). The frequency that each outcome was assessed and reported as altered in one or more exposure group across all included studies is shown in Table 4.

**TABLE 4:** Showing the number of cohorts that assessed each neurodevelopmental outcome and the number of cohorts reporting that those outcomes were altered.

Outcome area	Anti‐seizure medications						Other medications		Total	
	Grouped cohort studies^a^		Individual cohort studies		All studies		All studies			
	Assessed	Altered	Assessed	Altered	Assessed	Altered	Assessed	Altered	Assessed	Altered
**Cognitive development/Functioning**										
Infant global development	9	7	20	11	29	18	7	3	36	21 (58.3%)
IQ/Intellectual functioning	13	11	17	10	30	21	8	4	38	25 (65.7%)
Language development	12	6	9	4	21	10	4	1	25	11 (44.0%)
Memory	6	5	3	1	9	6	2	1	11	7 (63.6%)
Attention	5	4	2	2	7	6	1	1	8	7 (87.5%)
Executive functioning	4	3	0	0	4	3	1	0	5	3 (60.0%)
Processing speed	3	1	1	1	4	2	2	1	6	3 (50.0%)
Visuo‐spatial skills	6	3	2	0	8	3	3	2	11	5 (45.5%)
Learning disability diagnosis	4	3	0	0	4	3	1	0	5	3 (60.0%)
Learning difficulty diagnosis	1	0	1	0	2	0	0	0	2	0 (0%)
**Motor Development**										
Fine or Gross motor skills	10	7	9	2	19	9	1	0	20	9 (45.0%)
Dyspraxia	0	0	1	0	1	0	0	0	1	0 (0%)
**Behavioral/Emotional Functioning**										
Behavior problems	11	5	10	4	21	9	4	1	25	10 (40.0%)
Adaptive behavior	3	3	0	0	3	3	1	1	4	4 (100%)
Social skills	7	4	1	1	8	5	1	1	9	6 (66.7%)
Emotional Regulation/Mood Difficulties	4	2	3	3	7	5	5	4	12	9 (75.0%)
**Neurodevelopmental disorders**										
Attention deficit hyperactivity disorders	9	5	7	4	16	9	1	1	17	10 (58.8)
Autistic Spectrum Disorders	7	6	9	6	16	12	1	0	17	12 (70.6%)
Other/General neurodevelopmental disorders	3	3	1	1	4	4	0	0	4	4 (100%)
**Educational Outcomes**										
Rates of specialist educational need	4	2	5	2	9	4	0	0	9	4 (44.4%)
Examination results/School performance	1	1	3	3	4	4	1	0	5	4 (80.0%)
Other (e.g., Profession)	0	0	1	0	1	0	0	0	1	0 (0%)

^a^ Cohorts with multiple publications.

The results in Table 4 demonstrate that when neurodevelopmental outcomes are investigated for medication exposures with physical teratogenic signatures, there are high levels of neurodevelopmental alterations. For studies including global cognitive functioning outcomes, 58% found altered outcomes in infancy, with 66% of investigating studies finding an association with impaired intellectual functioning in older children. There were also high levels of alterations to other cognitive skills such as memory (64%), attention (88%), and executive functioning (60%). Social and emotional/mood difficulties were also reported in 67% and 75% of investigating studies respectively. Clinical diagnoses such as ADHD (59%) and ASD (71%) were also found to be higher in one or more of the medications included. Specific details regarding the neurodevelopmental outcomes assessed by each cohort are provided in Supplementary Tables [Link], [Link].

#### 3.1.3 Methodological Features of Cohorts Reporting Altered Outcomes

Key methodological features of cohorts that reported poorer neurodevelopmental outcomes are presented in Table 5 and are summarized for key outcomes below. This provides a broad indication of the quality and reliability of the data produced by these studies, informing the level of confidence that can be placed in the results. Across all included medications, 86.6% of cohorts reporting poorer neurodevelopmental outcomes included at least one comparison group, 57.8% controlled/corrected for at least one covariate, and 29.8% involved assessment by individuals who were blinded to exposure status. 68.1% of these studies were prospective, with 83.4% using primary data collection methods.

**TABLE 5:** Proportion of studies reporting altered neurodevelopmental outcomes that include key methodological features.

	Altered outcomes (N)	Age range	Comparison group (Yes)	Blinded assessment (Yes)	Covariates included (Yes)	Prospective (Yes)	Primary data (Yes)
Infant Global Development	21	2 months – 29 years	71.0%	33.3%	42.9%	81%	85.7%
IQ/Intellectual functioning	25	5 months −37 years	76.0%	60.0%	52.0%	68%	100%
Autistic Spectrum Disorders (inc. traits, behaviors etc.)	12	1–37 years	75.0%	0.0%	58.3%	66.70%	66.7%
Attention Deficit/Hyperactivity Disorders	10	3–37 years	90.0%	20.0%	70.0%	70%	70%
Attention	7	5–13 years	100.0%	50.0%	50.0%	83%	100%
Executive Functioning	3	3–7 years	100.0%	42.9%	42.9%	71%	100%
Memory	7	5–51 years	100.0%	57.1%	57.1%	71.43%	100%
Language	11	7 months – 39 years	82.0%	36.4%	72.7%	72.73%	81.8%
Learning disabilities	3	6 years	100.0%	0.0%	100.0%	100%	0%
School performance	4	7–18 years	100.0%	0.0%	50.0%	75%	25.0%
Additional Educational Needs	4	4–37 years	100.0%	25.0%	75.0%	25%	100%
Emotion Regulation/Mood Difficulties	9	< 1–51 years	89.0%	11.1%	33.3%	44%	67%
Social skills	6	7 months – 51 years	100.0%	33.3%	83.3%	66.67%	100%
Behavioral problems	10	21 months – 51 years	90.0%	0.0%	40.0%	50%	70.0%
Motor Skills (Fine and Gross)	9	3 months – 7 years	100.0%	22.2%	77.8%	89%	100%
Neurodevelopmental disorders (other)	4	4–37 years	100.0%	25.0%	75.0%	75%	50%
Adaptive behavior	4	3–16 years	75.0%	25.0%	75.0%	50%	100%
Processing Speed	3	3–51 years	100.0%	0.0%	33.3%	0%	100%
Spatial abilities	5	6–21 years	100.0%	40.0%	80.0%	80%	100%
**Weighted Mean**	**N/A**	**N/A**	**86.6%**	**29.8%**	**57.8%**	**68.1%**	**83.4%**

#### 3.1.4 Covariates Related to Outcomes

Covariates were included in analyses in 54% of studies investigating ASM exposure (Grouped Cohorts = 76%, Individual Cohorts = 45%), but in just 9% of studies investigating exposure to other teratogenic medications. The most common maternal factors that were reported to be significantly associated with neurodevelopmental outcomes across all cohorts were IQ (*n* = 12), education (*n* = 7), and socio‐economic status (*n* = 5). Other significant maternal factors included breastfeeding, maternal seizures, and maternal age (all *n* = 3). In terms of infant factors, gestational age (*n* = 5), age at assessment (*n* = 4), birthweight (*n* = 4) and sex (*n* = 3) were most commonly associated with outcomes, alongside outcome data from other child measures (*n* = 4).

#### 3.1.5 Dose and Timing Effects

Dose‐dependent effects were formally investigated in 22 cohorts, with 17 cohorts finding significant associations between at least one included exposure and at least one investigated outcome. All but one of the dose investigations were carried out in ASMs. Valproate was the most commonly assessed exposure (*n* = 17), followed by carbamazepine (*n* = 8), phenobarbital (*n* = 3), phenytoin (*n* = 2), topiramate (*n* = 2) and methimazole (*n* = 1).

Investigations into the effect of timing of exposure were only reported for eight cohorts, five of which reported that the window of exposure was a significant factor in neurodevelopmental outcomes. All but one of the cohorts that studied timing/duration effects were carried out in ASMs. Exposures included valproate (*n* = 6), carbamazepine (*n* = 2), topiramate (*n* = 2), phenobarbital (*n* = 1), and diethylstilbestrol (*n* = 1).

### 3.2 Case Series

One hundred and twenty‐six case series (*n* = 1 to 56 children across reports) were identified and included in this review, with many including case reports on multiple individuals. Sixty‐eight series (54%) reported exposure to ASMs (Abbas and Firdaus 2013; al‐Shammri et al. 1992; Alessandri et al. 2010; Ardinger et al. 1988; Arora et al. 2018; Barr Jr. et al. 1974; Bescoby‐Chambers et al. 2001; Carrim et al. 2007; Carter and Stewart 1989; Chessa and Iannetti 1986; Chitayat et al. 1988; Christianson et al. 1994; Clay et al. 1981; Cole et al. 2009; Dabee et al. 1975; DiLiberti et al. 1984; Fisher and Braddock 2001; Gigantelli et al. 2000; Godbole et al. 1999; Grech and Vella 1999; Hanson and Smith 1975; Hockey et al. 1996; Howe et al. 1995; Hoyt and Billson 1978; Huot et al. 1987; Iype et al. 2008; Jackson et al. 2020; Kalim and Reardon 2021; Karbhari Pawar and Zawar 2019; Khetarpal et al. 1999; Kikuchi 2016; Kozma 2001; Krauss et al. 1984; Kunz et al. 2013; Liguori and Cianfarani 2009; Loughnan et al. 1973; Malm et al. 2002; Massa et al. 1987; McMahon and Braddock 2001; Mutlu‐Albayrak et al. 2017; Okada et al. 1995; Ozkinay et al. 2003; Pandya and Jani 2000; Pearl et al. 1984; Phelan et al. 1982; Rybalko et al. 2019; Sabry and Farag 1996; Santos de Oliveira et al. 2006; Schorry et al. 2005; Seeler et al. 1979; Seip 1976; Shah et al. 2014; Sherman and Roizen 1976; Singh et al. 2016; Sparla et al. 2017; Speidel and Meadow 1972; Taylor et al. 1980; Thisted and Ebbesen 1993; Van Houtte et al. 2014; Verhoeven et al. 2009; Vestermark and Vestermark 1991; Waziri et al. 1976; Wester et al. 2002; Williams et al. 2001; Williams and Hersh 1997; Yalcikaya et al. 1997; Yang et al. 1978; Zellweger 1974), 17 (13%) to anticoagulants (Agarwal and Phadke 2013; Anonymous 1976; Basu et al. 2016; Bian et al. 2012; Collins et al. 1977; Holzgreve et al. 1976; Kaplan 1985; Khan 2007; Pati and Helmbrecht 1994; Pettifor and Benson 1975; Raghav and Reutens 2007; Ruthnum and Tolmie 1987; Safra et al. 2023; Sherman and Hall 1976; Silveira et al. 2015; Simonazzi et al. 2008; van Driel et al. 2002), 14 (11%) to antithyroid medication (Barbero, Ricagni et al. 2004; Bowman et al. 2012; Clementi et al. 1999; Ferraris et al. 2003; Foulds et al. 2005; Goel and Dudding 2013; Greenberg 1987; Gripp et al. 2011; Kalb and Grossman 1986; Martin‐Denavit et al. 2000; Mujtaba and Burrow 1975; Myers and Reardon 2005; Valdez et al. 2007; Washio 2020), 12 (10%) to immunosuppressants (Anderka et al. 2009; Ang et al. 2008; Bawle et al. 1998; Buckley et al. 1997; Del Campo et al. 1999; Delatycki 2005; Kozlowski et al. 1990; Lin et al. 2011; Marzec et al. 2021; Milunsky et al. 1968; Perez‐Aytes et al. 2008; Wheeler et al. 2002), nine (7%) to oral retinoids (Adam et al. 2007; Autret‐Leca et al. 2010; Barbero, Lotersztein et al. 2004; Ishijima and Sando 1999; Kritzinger and Steenkamp 2006; Lott et al. 1985; Rappaport and Knapp 1989; Rizzo et al. 1991; Van Abel et al. 2010), four (3%) to misoprostol (Coelho et al. 2000; Gonzalez et al. 1993; Rosa et al. 2007; Vendramini‐Pittoli et al. 2013), and two (2%) to thalidomide (Stromland et al. 1994; Vianna et al. 2013).

#### 3.2.1 Neurodevelopmental Outcomes Reported in Case Series

Impaired neurodevelopmental outcomes reported in single case reports and case series are stratified into two groups: ASMs and Other Medications. Table 6 shows the number of case series that included a report of each neurodevelopmental outcome and the number of those which reported altered neurodevelopmental outcomes. Note that some case reports/series describe multiple cases and are counted as a single report for the purpose of this summary.

**TABLE 6:** Number of case reports/series reporting each neurodevelopmental outcome, and the number reporting that those outcomes were altered.

Neurodevelopmental outcomes		Antiseizure medications (*n* = 68)		Other medications (*n* = 58)		Overall (*n* = 126)	
		Reports	*N* ^a^	Reports	*N*	Reports	*N*
Infant Global Development	Total Case Series	47	104	49	184	96	288
	Altered Outcomes^b^	38	55	25	69	63	124
IQ/Intellectual functioning	Total Case Series	26	70	12	71	38	141
	Altered Outcomes	16	30	7	24	23	54
Autistic Spectrum Disorders	Total Case Series	7	21	1	5	8	26
	Altered Outcomes	7	15	1	4	8	19
Attention Deficit/Hyperactivity Disorder	Total Case Series	2	5	0	N/A	2	5
	Altered Outcomes	2	3	0	N/A	2	3
Attention	Total Case Series	3	10	1	1	4	11
	Altered Outcomes	3	3	1	1	4	4
Executive Functioning	Total Case Series	1	3	0	N/A	1	3
	Altered Outcomes	1	2	0	N/A	1	2
Memory	Total Case Series	0	N/A	0	N/A	0	N/A
	Altered Outcomes	0	N/A	0	N/A	0	N/A
Language	Total Case Series	35	82	13	45	48	127
	Altered Outcomes	33	54	11	11	44	65
Learning disabilities	Total Case Series	7	22	4	34	11	56
	Altered Outcomes	7	21	3	6	10	27
School performance/Additional educational needs	Total Case Series	8	21	4	6	12	27
	Altered Outcomes	6	8	2	2	8	10
Psychiatric diagnoses/mental health	Total Case Series	0	N/A	0	N/A	0	N/A
	Altered Outcomes	0	N/A	0	N/A	0	N/A
Social skills	Total Case Series	4	14	0	N/A	4	14
	Altered Outcomes	4	4	0	N/A	4	4
Emotional/behavioral problems	Total Case Series	14	39	2	4	16	43
	Altered Outcomes	11	16	0	0	11	16
Motor Skills	Total Case Series	28	52	15	37	43	89
	Altered Outcomes	22	30	7	16	29	46
Neurodevelopmental disorders (other)	Total Case Series	0	N/A	0	N/A	0	N/A
	Altered Outcomes	0	N/A	0	N/A	0	N/A
Adaptive behavior	Total Case Series	3	8	0	N/A	3	8
	Altered Outcomes	3	6	0	N/A	3	6
Sensory processing/Processing speed	Total Case Series	0	N/A	0	N/A	0	N/A
	Altered Outcomes	0	N/A	0	N/A	0	N/A
Spatial abilities	Total Case Series	0	N/A	0	N/A	0	N/A
	Altered Outcomes	0	N/A	0	N/A	0	N/A

^a^ Total number of participants or patients included in case series.

^b^ Total number of case series that reported altered each neurodevelopmental outcome.

Like the empirical studies, there was a high proportion of case series that reported on neurodevelopmental outcomes that were altered. The most reported neurodevelopmental outcomes (altered or unaltered) across all included case series were infant global development (*n* = 96, 76%), language development (*n* = 48, 38%), motor skills (*n* = 43, 34%), and IQ/intellectual functioning (*n* = 38, 30%). The frequency that each outcome was assessed and reported as altered across all included case series is shown in Table 6.

### 3.3 Timing of Case Studies Versus Empirical Reports

**FIGURE 3:**
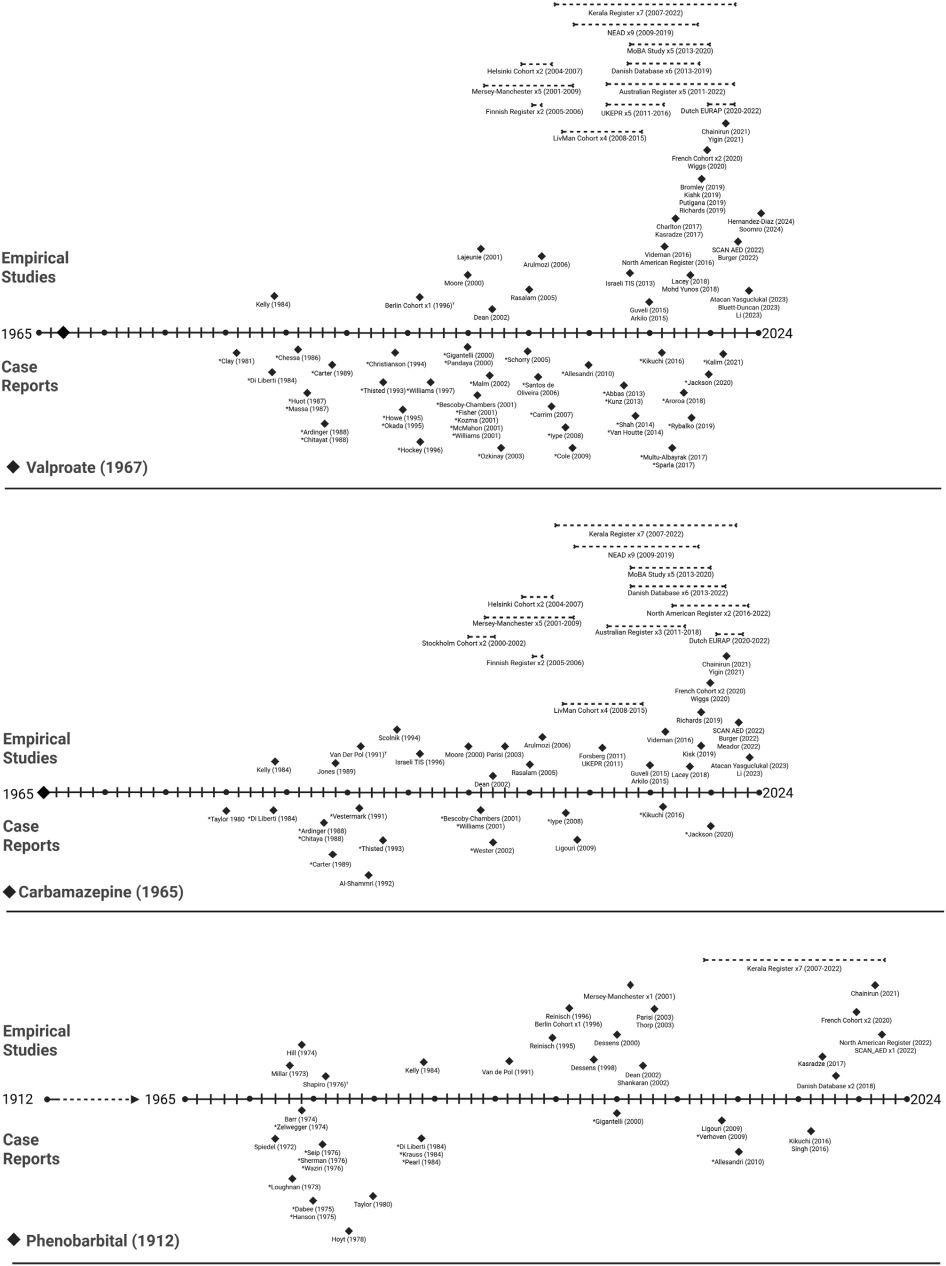
Timelines showing the market authorisation date and timing of case series and empirical studies included in this review that report neurodevelopmental outcomes for sodium valproate, carbamazepine and phenytoin. Ŧ = the first empirical study to include formal analysis and a comparator group; * = case studies reporting a significant neurodevelopmental impairment; ♦ = cohort results reported in a single publication for this medication; > − <= cohort results reported in multiple publications for this cohort, showing the period of time over which results are reported.

**FIGURE 4:**
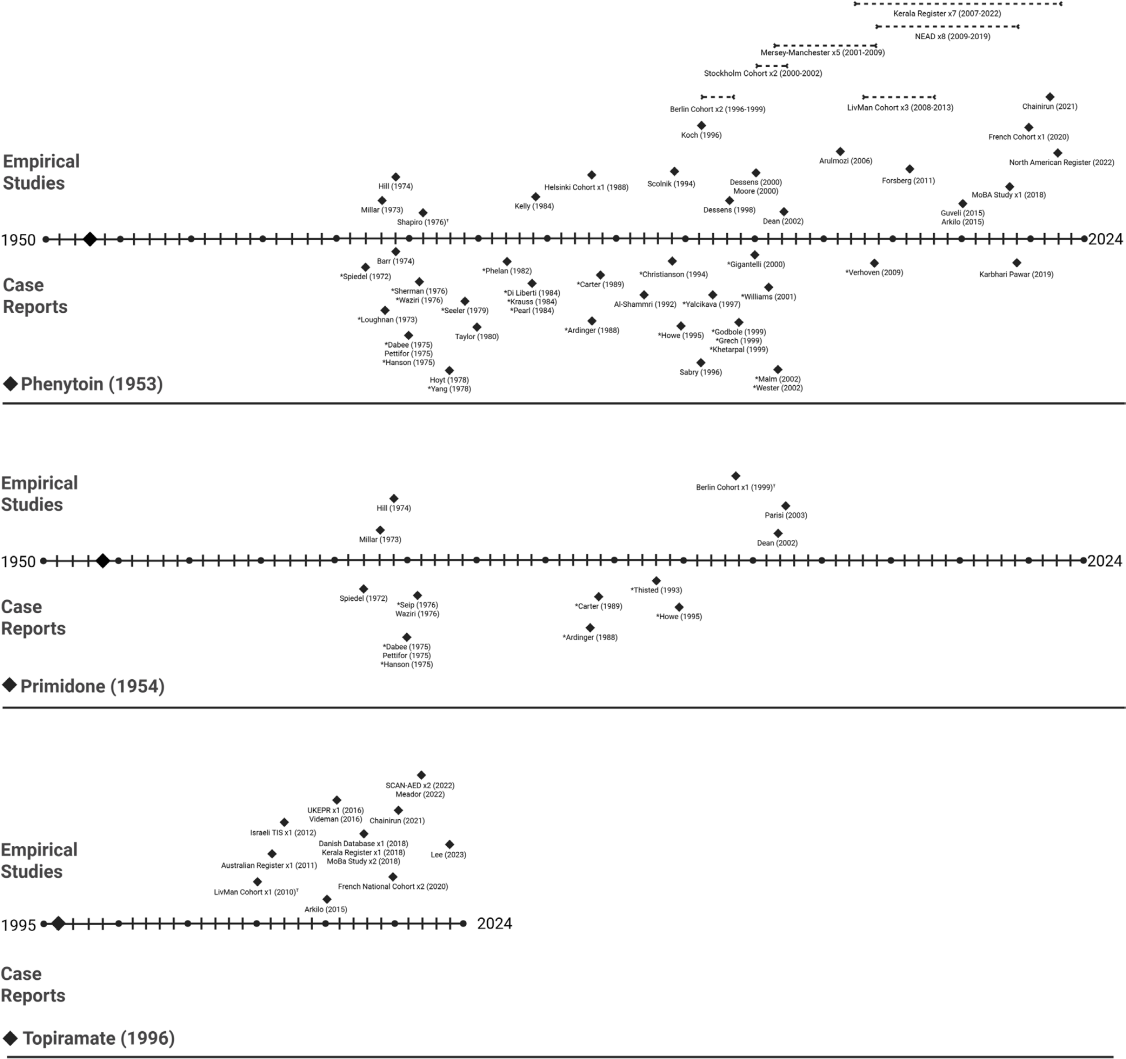
Timelines showing the market authorisation date and timing of case series and empirical studies included in this review that report neurodevelopmental outcomes for phenobarbital, phenytoin and topiramate. Ŧ = the first empirical study to include formal analysis and a comparator group; * = case studies reporting a significant neurodevelopmental impairment; ♦ = cohort results reported in a single publication for this medication; > − <= cohort results reported in multiple publications for this cohort, showing the period of time over which results are reported.

**FIGURE 5:**
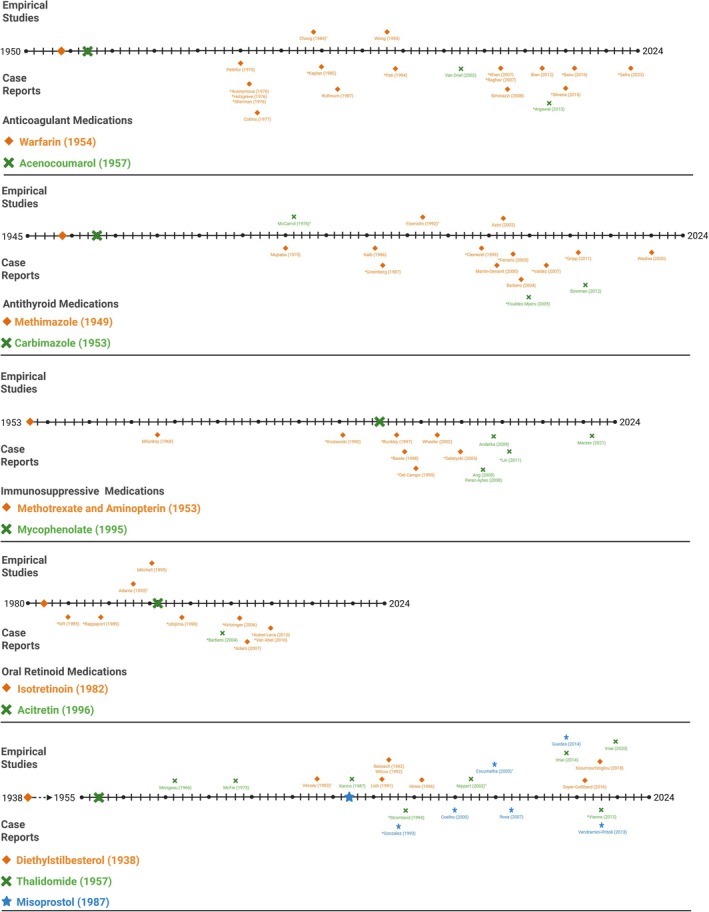
Timelines showing the market authorisation date and timing of case series and empirical studies included in this review that report neurodevelopmental outcomes for non‐antiseizure medications included in this review. Ŧ = the first empirical study to include formal analysis and a comparator group; * = case studies reporting a significant neurodevelopmental impairment; ♦, X , 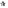 = cohort results reported in a single publication for the medication indicated in the key on each timeline.

Figures 3, 4, 5 provides a visual representation of the relative timing of the included case reports and empirical studies for each medication. The figures demonstrate the time from when each medication was first authorized for use and the first reports of neurodevelopmental outcomes (positive or negative) for that medication. To aid interpretation, case studies which reported an impaired neurodevelopmental outcome are marked with an asterisk. This is not intended to insinuate causality, due to known biases in adverse event reporting, but to demonstrate whether consistent risk signals were followed up with formal, empirical investigations. For each medication, the earliest reported authorization date was used, although this was challenging to ascertain in certain cases (see Supplementary Table 7 for source information). The mean time between licensing of medications subsequently shown to be structural teratogens and the first case report including any mention of neurodevelopment is 23 years (Range: 3–60 years). The mean time between medication licensing and the first empirical study that included a comparison group and conducted formal data analysis is 33 years (Range: 11–64 years).

For certain, medications there is a relative balance in the distribution and number of case reports and empirical studies, although generally there is considerable delay between case reports and empirical studies. This is primarily the case for the ASMs. Phenobarbital is a good example, with a flurry of case reports from 1972 to 1976 with a focus on empirical studies emerging from 1992 onwards. Topiramate, for which there are no case reports, is an exception to this pattern. Other medications tend to be weighted toward case reports, with very few empirical studies being conducted for non‐ASMs. The mean time between the first adverse case report and the first empirical study was 9.8 years (Range: 4–24 years) in the non‐ASM grouping. The exception being carbimazole, whereby a single empirical study was conducted 28 years before the first published case report.

## 4 Discussion

Eighty‐one empirical cohorts were identified that investigated neurodevelopmental outcomes following exposure to medications known to be associated with structural teratogenicity.

This review has a number of concerning findings. The first is that many medications with known structural teratogenic potential have received little or no investigation for their potential additional impact on brain development and later altered neurodevelopmental trajectories. Twenty‐four medications were identified by experts as having structural teratogenic potential, and empirical studies had been conducted in only 13 (54%) of these medications. When investigated, the rate of neurodevelopmental deviations present alongside physical teratogenic risk was high (77%), although it should be noted that the methodology and sensitivity of studies to infer causality varied significantly, as discussed below.

The second concerning finding is the substantial time latency between a medication being authorized for use and the first empirical study. This stood at an average of 33 years (range: 11–64 years). This is likely to be a conservative estimate as it refers to known structural teratogens only and excludes medications with physical teratogenic potential with no existing neurodevelopmental follow‐up. Previously, the time between post‐market authorization and a more precise safety classification was estimated to be 27 years, but this is largely based on major congenital anomaly risk (Adam et al. 2011). Strikingly, in the case of neurodevelopment, the 33 years is not the time taken to accumulate sufficient evidence for a precise risk classification, but the time elapsed before the process of accumulating empirical evidence even begins.

There has been a more significant focus in the literature toward investigating neurodevelopmental effects in ASM exposed cohorts of children compared to all other medications. Where investigations have been carried out, this review found that a range of cognitive, social, behavioral, and motor outcomes were altered across a number of medications and supports the call from a recent EWG (Bromley, Bickle Graz et al. 2023) that a broad range of outcomes must be investigated across a complementary set of studies to provide a comprehensive investigation of potential neurodevelopmental risk. The heavy weighting of ASM studies may be in part due to the known physical effects of certain medications in this class (e.g., valproate) (Bromley et al. 2014; Clayton‐Smith et al. 2019), which led to the establishment of a network of pregnancy registers that have provided access to cohorts of pregnant women and their children (Knight et al. 2023; Thomas, Salim et al. 2022). While it is encouraging to see that this finding may have resulted in somewhat improved investigation in ASM exposures, many ASMs remain without evidence, and it is apparent that this enthusiasm has not translated into other medication classes. Even where there is known structural teratogenic risk, indicating potentially heightened risk for neurodevelopment, the number of high‐quality empirical studies remains unacceptably low.

A clear delineation in the methodology utilized for ASM studies and other medications was also apparent. The EWG developed a set of consensus guidelines for optimal neurodevelopmental follow‐up of teratogenic exposure (Bromley, Bickle Graz et al. 2023). When specific medication classes were rated against the criteria within the scope of this review, the total evidence base for ASMs scored consistently higher than for the other medications assessed (Table 4). Optimal design features such as prospective recruitment, the use of blinded assessments, investigations of dose and timing effects, the use of appropriate comparison groups, and the inclusion of relevant covariates (e.g., maternal indication, family history of neurodevelopmental difficulties, and socioeconomic status), key for disentangling complex causal pathways, were more common in ASM studies. While it is apparent that neurodevelopmental investigations require methodological improvements across the board, ASM investigations do appear to be significantly more developed than investigations of other known teratogenic medications. This is highlighted by the use of newer approaches to collecting data in ASM studies, such as the use of population health databases (Bjork et al. 2022; Blotiere et al. 2020; Christensen et al. 2019; Wiggs et al. 2020), that are absent in investigations of other medications and demonstrates the concerning lack of progress being made in wider pregnancy pharmacovigilance.

Clearly, improvements are needed in the design and optimization of investigations. The role of the dose and timing of exposure is a central tenet of teratology (Vorhees 1986; Adams et al. 2000) however, dose was formally investigated in just 25% of included studies across all medications, while gestational timing was investigated in just 9%. Significant dose‐dependent effects were found in 15 out of 19 cohorts across a wide array of outcomes, including IQ, infant global development, motor skills, processing skills, executive function, language, and clinical disorders, demonstrating the sensitivity of neurodevelopment to dose effects and the importance of including this factor in future neurodevelopmental studies if the literature is to accurately inform regulatory and clinical decision making.

Case series that report neurodevelopmental outcomes were identified for sixteen of the twenty‐four included teratogens. These series reported a range of typical and altered outcomes and, as with the empirical studies, no conclusions are drawn regarding the neurodevelopmental impact of specific medications as part of this review. However, the pattern of reporting demonstrate which outcomes are more frequently reported using this methodology as well offering key insights regarding the expectations and reality of how pregnancy pharmacovigilance is functioning. Pregnancy pharmacovigilance has historically relied upon “*astute clinician*” *and* “adverse event reporting” approaches to identify potential risk signals to direct further investigation (Carey et al. 2009). Inspection of Figure 3 indicates that this model has worked to a certain degree in the older ASMs, as case reports are clustered toward the beginning of the timeline, and empirical studies are clustered toward the end. However, in the non‐ASM medications, there is no clear pattern of empirical studies following on from case reports. For example, adverse neurodevelopmental events have been reported in several case reports for both methimazole and methotrexate, but there are little or no empirical investigations for these medications. Additionally, for newer medications, such as topiramate, there are few or no case studies. This may indicate a lack of risk for those medications but could also indicate a failure of this system to identify neurodevelopmental effects as appears to be the case with topiramate where empirical studies indicate increased neurodevelopmental risk despite the lack of signal from case reports.

Notably, the majority of studies took place in high‐income countries. While this review has revealed the significant global challenges regarding neurodevelopmental follow‐up in pregnancy pharmacovigilance, the paucity of research in low‐ and middle‐income countries is particularly stark. There is a clear need for investment in culturally sensitive pregnancy pharmacovigilance infrastructure in resource‐constrained settings, both to ensure the recruitment of more diverse populations but also to address medication safety questions specific to those contexts (Shafi et al. 2024; Kiguba et al. 2023).

### 4.1 Implications

The issues raised in this review are symptomatic of the historic lack of prioritization of neurodevelopmental outcomes in pregnancy pharmacovigilance initiatives (Charlton and Vries 2012; Roque Pereira et al. 2022). While there is an increased recognition of the importance of neurodevelopmental follow‐up of medication exposure by regulators worldwide, as evidenced by recent updates to pharmacovigilance guidelines (European Medicines Agency 2019; U.S. Food and Drug Administration 2019), there remains little guidance on how and when these investigations should be carried out. The recent EWG regarding neurodevelopmental follow‐up of potentially teratogenic medications goes some way to rectifying this (Bromley, Bickle Graz et al. 2023). A set of consensus‐based recommendations regarding how and when investigations should be prioritized, optimal methodological approaches, and guidance on reporting outcomes is provided and can be a useful tool for regulators, pregnancy pharmacovigilance initiatives and market authorization holders in developing high quality investigations.

The adoption of these recommendations specifically, and the improvement of pregnancy pharmacovigilance more generally, can only advance in the context of an improved funding model, whereby regulatory authorities, market authorization holders, and other funding bodies take a more systematic and proactive approach. Higher costs and limited access to funding for neurodevelopmental follow‐up have been highlighted as primary barriers to conducting high quality research in this area (Bromley, Bickle Graz et al. 2023), and a number of calls have been made for a more sustainable, systematic funding approach (European Medicines Agency 2020; Meador and Loring 2016). It is noted here that most investigations were funded by research grants which are never certain and lead to a situation whereby the science is being led by the availability of the funding. A concrete step toward resolving these funding challenges would be for regulatory authorities to mandate neurodevelopmental post‐authorisation safety studies, unlocking consistent funding from market authorization holders. The gaps in knowledge across a broad array of known structural teratogens identified by this review adds further weight to this call for an improved funding model. While the costs of optimal neurodevelopmental follow‐up are relatively high, the cost of inaction regarding neuroteratogenic exposure can be considerably higher, both to the individual and to society. This is illustrated by a recent case in which two settlements totaling €18 million were awarded for two children who were exposed to valproate during pregnancy (O'Donnell 2023). An estimated 20,000 children are estimated to have been affected by valproate exposure in the UK alone (Cumberlege 2020). Even without litigation or redress, the lifetime cost to support 20,000 individuals with neurodevelopmental difficulties is arguably higher than the cost to fund timely investigations (Buescher et al. 2014; Centre for Disease Control and Prevention 2004).

### 4.2 Limitations

The scoping nature of this review means that there are several limitations in how the data should be interpreted. We have only provided a broad overview of altered neurodevelopmental outcome assessment for known structural teratogenic medicines, analyzed as two broad categories of ASMs and non‐ASMs. Delineation of the neurobehavioral signature for teratogens which impact the brain is not assessed at individual exposure level. An accurate understanding of the clinical implications of any observed altered neurodevelopmental outcomes requires further detailed and systematic review. This was outside the scope of the current review and so we have intentionally avoided speculating about individual medication risk/safety profiles. There was no formal quality or risk of bias assessment for included studies. Again, this was beyond the current scope, but we believe the detailed assessment of different methodological aspects of each study serves as a sufficient proxy. In grouping all ASMs together, it is possible that the strengths of the research regarding this medication class have been magnified. For example, medications such as valproate and carbamazepine have received far more attention than topiramate and primidone. Therefore, the current findings should not be considered an overall endorsement of the state of pregnancy pharmacovigilance across all ASMs. Finally, limiting the included studies to those of known human teratogens limits the conclusions we can provide generally about neurodevelopmental assessment for teratogens that do not have a strong physical signature.

## 5 Conclusion

These findings do not speak to a pharmacovigilance system that is functioning efficiently to quickly identify and ameliorate neurodevelopmental risk, even for the medications with identified structural teratogenic risk. Despite a high proportion of known physical teratogens additionally having an association with altered neurodevelopmental outcomes and the substantial lifetime burden of such outcomes to the individual and society, the timelines remain too long and funding too precarious.

## Author Contributions

M. Bluett‐Duncan: study screening, data extraction, analysis, writing – original draft, review and editing. R.L. Bromley: conceptualization, protocol development, study screening, analysis, writing – review and editing. S. Khanom: study screening, data extraction, analysis, writing – review and editing. J.L. Richardson: conceptualization, protocol development, study screening, data extraction, writing – review and editing. M. Stellfeld, D. Mølgaard‐Nielsen, A. Cahoon, C. Jackson: protocol development, study screening, writing – review and editing. LMY: conceptualization, protocol development, writing – review and editing. J. Adams, M. Bluett‐Duncan, M. Berlin, J. Clayton‐Smith, V. Simms, U. Winterfeld: protocol development, writing – review and editing.

## Conflicts of Interest

The authors declare no conflicts of interest.

## Supporting information


**Supplementary Table 1.** Key methodological aspects of included cohorts with multiple study publications investigating exposure to ASMs.

**Supplementary Table 2.** Key methodological aspects of included cohorts with a single publication investigating exposure to ASMs.

**Supplementary Table 3.** Key methodological aspects of included cohorts with a single publication investigating exposure to other medications.

**Supplementary Table 4.** Neurodevelopmental outcomes assessed and reported to be significantly altered in ASM cohorts reporting multiple studies. Outcomes may have been measured and/or assessed in one or multiple studies within each cohort.

**Supplementary Table 5.** Neurodevelopmental outcomes assessed and reported to be significantly altered in ASM cohorts reporting single studies.

**Supplementary Table 6.** Neurodevelopmental outcomes assessed and reported to be significantly altered in cohorts reporting exposure to other medications.

**Supplementary Table 7.** Timing of approval and publications for each included medication.

## Data Availability

The data that support the findings of this study are available from the corresponding author upon reasonable request.
