# Supplementary material for: A Scoping Review of Human Teratogens and Their Impact on the Developing Brain: A Contribution From the ConcePTION Project
Source: Birth Defects Res. 2025 Sep 17;117(9):e2497. doi: 10.1002/bdr2.2497 (PMC12442749; doi:10.1002/bdr2.2497)
Supplement: Supplementary file 1 — Supplementary Table 1. Key methodological aspects of included cohorts with multiple study publications investigating exposure to ASMs. [file BDR2-117-e2497-s004.docx]

Supplementary Table 1: Key methodological aspects of included cohorts with multiple study publications investigating exposure to ASMs.

| **Cohort** | **Setting** | **Design** | **ASMs** | **Studies** | **Age range** | **Exposure Data** | **Outcome Data** | **Comparison Group(s)** | **Significant Confounders or Covariates** |
| --- | --- | --- | --- | --- | --- | --- | --- | --- | --- |
| Australian Pregnancy Register | Australia | - Prospective Observational Cohort - Community Setting - Primary data, directly collected for this study. | CBZ  VPA  TPM | Wood 2015  Nadebaum 2011a†  Nadebaum 2011b  Barton 2018  Honybun 2021 | 6-8 years | Maternal report | Researcher (blinded); Parent | Other medication exposed;  Unexposed, general population. | - Folic Acid (1st trimester) - Marijuana use - One or more seizures during pregnancy - Convulsive seizure(s) - Maternal IQ - SES - Language outcomes - Child age - Child sex |
| Berlin Cohort | Germany | - Prospective Observational Cohort - Hospital setting - Primary data, directly collected for this study | PB  PHT  VPA PRM | Koch 1996†  Koch 1999 | 6-19 years | Hospital notes or records | Researcher | Unexposed, general population | None Reported^‡^ |
| Danish Health Database | Denmark | - Prospective Observational Cohort - Population Database - Secondary data, routinely collected | CBZ, PB, TPM, VPA, | Bech 2018  Christensen 2013†  Christensen 2019  Daugaard 2020  Elkjaer 2018  Ren 2022 | 6-16 years | Pharmacy records | Education System;  Health Professional | Unexposed, disease matched;  Other medication exposed;  Unexposed, general population. | - Preterm birth - Low birth weight - Child birth year |
| Dutch EURAP Study | Netherlands | - Prospective Observational Cohort - Community setting - Primary data, directly collected for this study. | CBZ, VPA, | Huber-Mollema 2019†  Huber-Mollema 2020 | 6-7 years | Maternal report | Parent; Researcher | Other medication exposed;  Unexposed, general population | - Gestational ag, - Age of child - Maternal IQ - Maternal Education |
| Finnish Pregnancy Register | Finland | - Prospective Observational Cohort - Community setting - Primary data, directly collected for this study. | CBZ, VPA | Eriksson 2005†  Viinikainen 2006 | 6-13 years | Hospital notes or records | Health Professional; Researcher (blinded); Education System | Unexposed, disease-matched;  Other medication exposed. | N/A^§^ |
| French National Cohort | France | - Prospective Observational Cohort - Population Database - Secondary data, routinely collected | CBZ  PB  PHT  TPM  VPA | Blotiere 2020†  Coste 2020 | 4 years | Hospital notes or records | Health Professional | Unexposed, general population;  Other medication exposed | Maternal mental illness |
| Helsinki University Central Hospital | Finland | - Prospective Observational Cohort - Hospital setting - Primary data, directly collected for this study. | CBZ, VPA | Gaily 2004†  Kantola-Sorsa 2007 | 5-11 years | Hospital notes or records | Researcher (blinded) | Unexposed, general population;  Other medication exposed. | N/A |
| Israeli TIS | Israel | - Prospective Observational Cohort - Community setting - Primary data, directly collected for this study. | CBZ  TPM  VPA | Ornoy 1996†  Rihtman 2012  Rihtman 2013 | 6m – 7 years | Maternal report | Parent; Researcher (blinded); Education System | Unexposed, general population | N/A |
| Kerala Pregnancy Register | India | - Prospective Observational Cohort - Community setting - Primary data, directly collected for this study. | CBZ, PB, PHT, TPM, VPA, | Asranna 2018  Gopinath 2015  Thomas 2007†  Thomas 2008  Unnikrishnan 2020  Thomas 2022a (IQ)  Thomas 2022b (DQ) | 12m – 21y | Maternal report | Researcher (blinded) | Unexposed, disease-matched;  Unexposed, general population;  Other medication exposed | - BMI - Maternal education - Maternal IQ - AED dosage - Paternal education - Number of AEDs |
| Liv-Man Epilepsy Cohort | UK | - Prospective Observational Cohort - Community setting - Primary data, directly collected for this study. | CBZ, PHT, TPM, VPA, | Baker 2015  Bromley 2008  Bromley 2010†  Bromley 2013 | 2m-6y | Maternal Report | Researcher (blinded)  Health Professional | Unexposed, general population;  Other medication exposed. | - Maternal IQ - SES - Gestational Age. - Gender |
| Mersey-Manchester Regional Epilepsy Clinics | UK | - Retrospective Observational Cohort - Hospital setting - Primary data, directly collected for this study. | CBZ, PB, PHT, VPA | Adab 2001†  Adab 2004  Kini 2006  Vinten 2005  Vinten 2009 | 6m-18y | Maternal report | Parent; Researcher (blinded) | Unexposed, disease matched;  Other medication exposed. | - Maternal age Maternal IQ - SES - Learning disability in 1st degree relative - Seizures during pregnancy - NART score - Child FSIQ - Maternal FSIQ - Generalised Epilepsy - Child Age |
| NEAD | USA & UK | - Prospective Observational Cohort - Community setting - Primary data, directly collected for this study. | CBZ  PHT VPA | McVearry 2009  Cohen 2011  Cohen 2013  Cohen 2019  Meador 2009†  Meador 2010  Meador 2011  Meador 2012  Meador 2013 | 2-6 years | Maternal report | Researcher (blinded); Parent; Teacher | Other medication exposed;  Unexposed, general population | - Child IQ, breastfeeding - Maternal IQ - Standardized AED dose - Gestational age - Site location. Maternal education - Folate use - Maternal age - AED group - Race/Ethnicity - Alcohol exposure |
| North American Register | USA | - Prospective Observational Cohort - Community setting - Primary data, directly collected for this study | CBZ  PHT PB  VPA | Deshmukh 2016†  Adams 2022 | 3-16.9 years | Maternal report | Parent; Researcher (blinded) | Unexposed, general population | - Maternal IQ |
|  |  |  |  |  |  |  |  |  |  |
| Norwegian MoBa Study | Norway | - Prospective Observational Cohort - Birth cohort linked with national registry data. - Primary data, directly collected for this study, and secondary routine data. | CBZ  TPM  VPA  PB  PHT | Husebye 2018  Husebye 2020  Veiby 2013a†  Veiby 2013b  Bjork 2018 | 6m-8y | Maternal report | Parent | Unexposed, general population;  Other medication exposed;  Unexposed, disease matched. | - Low birth weight - Maternal anxiety/depression - Continuous breastfeeding |
|  |  |  |  |  |  |  |  |  |  |
| SCAN-AED | Denmark, Finland, Iceland, Norway, and Sweden | - Prospective Observational Cohort - Population Database - Secondary Routine | CBZ  PB  TPM  VPA | Bjork 2022†  Dreier 2023 | 8-18 years | Hospital notes or records | Health Professional | Unexposed, general population.  Unexposed, disease matched. | None Reported |
| Stockholm Cohort | Sweden | - Prospective Observational Cohort - Hospital setting - Primary data, directly collected for this study. | CBZ, PHT | Wide 2000†  Wide 2002 | 9m-5y | Hospital notes or records | Researcher (blinded) | Unexposed, general population. | None Reported |
| UKEPR | UK | - Prospective Observational Cohort - Community setting - Primary data, directly collected for this study. | CBZ VPA  TPM | Cummings 2011†  Bromley 2016  Shallcross 2011  Shallcross 2014 | 3m-9y | Maternal report | Researcher (blinded) | Unexposed, disease matched;  Other medication exposed. | - Maternal age - Gender - SES - Maternal IQ - Maternal seizures - Child age at assessment - Child sex |

† = Primary study, ‡ None Reported = No covariates with significant association with outcome, § N/A = No covariates included in analysis. CBZ = Carbamazepine, PB = Phenobarbital, PHT = Phenytoin, PRM = Primidone, TPM = Topiramate, VPA = Valproate
