# Supplementary material for: A Scoping Review of Human Teratogens and Their Impact on the Developing Brain: A Contribution From the ConcePTION Project
Source: Birth Defects Res. 2025 Sep 17;117(9):e2497. doi: 10.1002/bdr2.2497 (PMC12442749; doi:10.1002/bdr2.2497)
Supplement: Supplementary file 2 — Supplementary Table 2. Key methodological aspects of included cohorts with a single publication investigating exposure to ASMs. [file BDR2-117-e2497-s002.docx]

Supplementary Table 2: Key methodological aspects of included cohorts with a single publication investigating exposure to ASMs.

| **Study** | **Setting** | **Design** | **ASMs** | **N** | **Age range** | **Exposure Data** | **Outcome Data** | **Comparison Group(s)** | **Significant Confounders or Covariates** |
| --- | --- | --- | --- | --- | --- | --- | --- | --- | --- |
| Arkilo 2015 | USA | - Retrospective Observational Cohort - Community setting - Primary data, directly collected for this study | CBZ  PHT  TPM  VPA | 62 | 2 years | Maternal report | Researcher | None | N/A^†^ |
| Arulmozhi 2006 | India | - Prospective Observational Cohort - Hospital setting - Primary data, directly collected for this study | CBZ  PHT  VPA | 60 | 2 years | Hospital notes or records | Researcher | Unexposed, general population | N/A |
| Atacan Yasguclukal 2023 | Turkey | - Retrospective Observational Cohort - Hospital Setting - Primary data, directly collected for this study | CBZ  VPA | 64 | 3m-18 years | Hospital notes or records | Parent | Unexposed, disease matched.  Other medication exposed. | None reported^‡^ |
| Bluett-Duncan 2023 | UK | - Retrospective Observational Cohort - Community Setting - Primary data, directly collected for this study | VPA | 146 | 7-37 years | Maternal Report | Parent | Unexposed, disease matched.  Unexposed, general population.  Other medication exposed. | N/A |
| Bromley 2019 | UK | - Retrospective Observational Cohort - Community setting - Primary data, directly collected for this study | VPA | 31 | 6-27 years | Maternal report | Researcher | Unexposed, general population. | None reported |
| Burger 2022 | South Africa | - Prospective Observational Cohort - Hospital - Primary data, directly collected for this study | CBZ  VPA | 112 | 10-20 weeks | Hospital records and maternal report | Researcher (blinded) | Other medication exposed | None Reported |
| Chainirun 2021 | Thailand | - Retrospective Observational Cohort - Hospital - Primary data, unclear if collected for this study | CBZ  PB  PHT  TPM  VPA | Not Reported | Not Reported | Not Reported | Not Reported | None | N/A |
| Charlton 2017 | UK | - Prospective Observational Cohort - Population Database - Secondary data from research database. | CBZ  VPA | 7480 | 6 years | Hospital notes or records | Health Professional | Unexposed, general population; Other medication exposed | Alcohol consumption |
| Dean 2002 | UK | - Retrospective Observational Cohort - Hospital - Secondary data, collected routinely for research. | CBZ  PHT  PB  PRM  VPA | 293 | 21 months - 39 years | Hospital notes or records | Health Professional; Education System | Unexposed, disease matched | N/A |
| Dessens 1998 | Netherlands | - Retrospective Observational Cohort - Community setting - Primary data, directly collected for this study | PB  PHT | 294 | N/A | Hospital notes or records | Researcher | Unexposed, general population | - Participant (exposed) IQ |
| Dessens 2000 | Netherlands | - Retrospective Observational Cohort - Hospital setting - Primary data, directly collected for this study | PB  PHT | 294 | N/A | Hospital notes or records | Researcher (blinded) | Unexposed, general population | N/A |
| Forsberg 2011 | Sweden | - Prospective Observational Cohort - Population Database - Secondary data, routinely collected | CBZ  PHT | 1,235 | 16 years | Hospital notes or records | Education System | Unexposed, general population | None reported |
| Gaily 1988 | Finland | - Prospective Observational Cohort - Hospital setting - Primary data, directly collected for this study | PHT | 226 | 5.5 years | Hospital notes or records | Researcher (blinded) | Unexposed, general population | N/A |
| Guveli 2015 | Turkey | - Retrospective Observational Cohort - Hospital setting - Primary data, directly collected for this study | CBZ  PB  PHT  VPA | 41 | 6-15 years | Maternal report | Researcher | Unexposed, disease-matched; Other medication exposed | N/A |
| Hernandez-Diaz 2024 | USA | - Prospective Observational Cohort - Population Database - Secondary Data, routinely collected | TPM  VPA | 4,292,539 | 8 years | Hospital notes or records | Health professional | Unexposed, disease matched  Other medication exposed | None reported |
| Hill 1974 | USA | - Prospective Observational Cohort - Hospital setting - Primary data, directly collected for this study | PB  PHT  PRM | 193 | 9, 12, 18, 21 or 24, and 36 months | Maternal report | Researcher (blinded) | Unexposed, general population | N/A |
| Jones 1989 | USA | - Prospective and retrospective components - Community setting - Primary data, directly collected for this study | CBZ | 43 | Varied | Maternal report | Health Professional; Researcher (non-blinded) | None | N/A |
| Kasradze 2017 | Georgia | - Prospective Observational Cohort - Community setting - Primary data, directly collected for this study | CBZ  PB  VPA | 100 | 36-72m | Hospital notes or records | Researcher (blinded) | Unexposed, general population | - Developmental milestones - Maternal IQ. |
| Kelly 1984 | USA | - Prospective Observational Cohort - Hospital setting - Primary data, directly collected for this study | CBZ  PB  PHT  VPA | 171 | Varied | Hospital notes or records | Researcher | Unexposed, disease-matched; Other medication exposed. | N/A |
| Kishk 2019 | Egypt | - Retrospective Case Control Study - Hospital setting - Primary data, directly collected for this study | CBZ  VPA | 80 | 5-16 years | Hospital notes or records | Researcher (blinded) | Unexposed, general population | - Maternal IQ |
| Lacey 2018 | UK | - Prospective Observational Cohort - Population Database - Secondary data, routinely collected | CBZ  VPA | 2196 | 7 years | Hospital notes or records | Education System | Unexposed, general population | N/A |
| Lajeunie 2001 | France | - Prospective Observational Cohort - Hospital setting - Primary data, directly collected for this study | VPA | 17 | 1-72 months | Maternal report | Researcher (non-blinded) | None | N/A |
| Li 2023 | China | - Prospective case-control study - Hospital setting - Primary data, directly collected for this study. | CBZ  TPM  VPA | 781 | ≤1 year | Maternal report | Researcher | Unexposed, disease matched  Other medication exposed | N/A |
| Meador 2021 | USA | - Prospective Observational Cohort - Hospital setting - Primary data, directly collected for this study | CBZ  TPM | 382 | 2 years | Maternal report | Researcher | Other medication exposed | - Maternal IQ - Maternal Education - Postpartum anxiety/depression - Folate Use - Breastfeeding - Ethnicity - Birthweight - Postpartum Sleep Quality |
| Millar 1973 | UK | - Prospective Observational Cohort - Hospital setting - Primary data, routinely collected | PB  PHT  PRM | 110 | N/A | Hospital notes or records | Health Professional | None | N/A |
| Mohd Yunos 2018 | Ireland | - Retrospective Observational Cohort - Hospital setting - Primary data, routinely collected | VPA | 29 | N/A | Hospital notes or records | Health Professional | None | N/A |
| Moore 2000 | UK | - Retrospective Observational Cohort - Hospital setting - Primary data, directly collected for this study | CBZ  PHT  VPA | 57 | 8 months - 16 years | Maternal report | Health Professional | None | N/A |
| Parisi 2003 | Italy | - Prospective Observational Cohort - Hospital setting - Primary data, directly collected for this study | CBZ  PB  PRM | 11 | 7 days, 4 & 13 weeks, 6, 9, 12 and 30 months. | Hospital notes or records | Researcher | None | N/A |
| Putignano 2019 | Italy | - Prospective Observational Cohort - Population Database - Secondary data, routinely collected | CBZ  VPA | 4208 | Up to 1 year | Pharmacy records | Health Professional | Unexposed, general population | N/A |
| Rasalam 2005 | UK | - Retrospective Observational Cohort - Hospital setting - Primary data, directly collected for this study | CBZ  VPA | 260 | 10 years | Maternal report | Health Professional | Unexposed, general population | N/A |
| Reinisch 1995 | Denmark | - Prospective Observational Cohort - Hospital setting - Primary data, directly collected for this study | PB | 267 | Study 1= 23 years Study 2 = 19 years | Hospital notes or records | Researcher (blinded) | Unexposed, general population | - SES - Unwanted Pregnancy |
| Richards 2019 | New Zealand | - Retrospective Observational Cohort - Population Database - Secondary data, collected routinely | CBZ  VP | 606 | 4 years | Pharmacy records | Health Professional | Unexposed, general population; Other medication exposed | None reported |
| Scolnik 1994 | Canada | - Prospective Observational Cohort - Hospital setting - Primary data, directly collected for this study | CBZ  PHT | 140 | 18-36 months | Maternal report | Researcher (blinded) | Unexposed, general population | None reported |
| Shankaran 1996 | USA | - Prospective Randomised Control Trial - Hospital setting - Primary data, directly collected for this study | PB | 127 | 12, 24 + 36 months | Hospital notes or records | Researcher | Unexposed, disease matched | N/A |
| Shankaran 2002 | USA | - Prospective Randomised Control Trial - Hospital setting - Primary data, directly collected for this study | PB | 436 | 18-22 months | Hospital notes or records | Researcher (blinded) | Unexposed, general population | - ICH - PVL - Maternal Education - Antenatal Steroid Use - Birth Weight |
| Shapiro 1976 | USA | - Prospective Observational Cohort - Hospital setting - Primary data, directly collected for this study | PB  PHT | 50,282 | N/A | Maternal report | Health Professional | Unexposed, general population; Other medication exposed | None reported |
| Soomro 2024 | Pakistan | - Prospective observational cohort - Hospital setting - Primary data, directly collected for this study | VPA | 210 | ≤4 years | Hospital notes or records | Health professional  Researcher | Unexposed, general population  Other medication exposed | None reported |
| Thorp 2003 | USA | - Prospective Randomised Control Trial - Secondary Research - Hospital setting - Secondary data, collected directly for another study. | PB | 299 | 7 years | Hospital notes or records | Researcher (blinded) | Unexposed, disease matched | - Maternal education - White race - Maternal smoking, Preterm premature rupture of membrane Premature labour Gestational age - Multiple gestation - 5 min apgar score |
| Van der Pol 1991 | Netherlands | - Retrospective Observational Cohort - Hospital - Primary data, directly collected for this study | CBZ  PB | 61 | 7-13 years | Hospital notes or records | Researcher (blinded) | Unexposed, disease matched | N/A |
| Videman 2016 | Finland | - Prospective Observational Cohort - Hospital setting - Primary data, directly collected for this study. | CBZ  TPM  VPA | 118 | 7 months | Hospital notes or records | Researcher (blinded) | Unexposed, general population | Maternal Education |
| Wiggs 2020 | Sweden | - Prospective Observational Cohort - Population Database - Secondary data, routinely collected | CBZ  VPA | 14,614 | 2-10 years | Hospital notes or records | Health Professional | Unexposed, general population | None reported |
| Yigin 2021 | Turkey | - Retrospective Observational Cohort - Hospital - Primary data, directly collected for this study | CBZ  VPA | 183 | Not reported | Hospital notes or records | Health Professional | Unexposed, general population | N/A |

† N/A = No covariates included in analysis. ‡ None Reported = No covariates with significant association with outcome. CBZ = Carbamazepine, PB = Phenobarbital, PHT = Phenytoin, PRM = Primidone, TPM = Topiramate, VPA = Valproate
