# Supplementary material for: A Scoping Review of Human Teratogens and Their Impact on the Developing Brain: A Contribution From the ConcePTION Project
Source: Birth Defects Res. 2025 Sep 17;117(9):e2497. doi: 10.1002/bdr2.2497 (PMC12442749; doi:10.1002/bdr2.2497)
Supplement: Supplementary file 3 — Supplementary Table 3. Key methodological aspects of included cohorts with a single publication investigating exposure to other medications. [file BDR2-117-e2497-s005.docx]

Supplementary Table 3: Key methodological aspects of included cohorts with a single publication investigating exposure to other medications.

| **Study** | **Setting** | **Design** | **Medication** | **N** | **Age Range** | **Exposure Data** | **Outcome Data** | **Comparison Group(s)** | **Significant Confounders or Covariates** |
| --- | --- | --- | --- | --- | --- | --- | --- | --- | --- |
| Imai 2014 | Japan | - Retrospective Observational Cohort - Hospital setting - Primary data, directly collected for this study | Thalidomide | 22 | 47-51 years | Hospital notes or records | Researcher | Unexposed, general population | N/A |
| Imai 2020 | Japan | - Retrospective Observational Cohort - Hospital setting - Primary data, directly collected for this study | Thalidomide | 51 | 54 years | Maternal Report | Self-report | None | N/A |
| Kanno 1987 | Japan | - Retrospective Observational Cohort - Hospital setting - Primary data, directly collected for this study | Thalidomide | 137 | 7-22 years | Hospital notes or records | Researcher | None | N/A |
| McFie 1973 | UK | - Retrospective Observational Cohort - Hospital setting - Primary data, directly collected for this study | Thalidomide | 54 | 7-10 years | Hospital notes or records | Researcher | None | N/A |
| Mongeau 1966 | Canada | - Retrospective Observational Cohort - Hospital setting - Primary data, directly collected for this study | Thalidomide | 34 | 32-41 months | Hospital notes or records | Researcher | None | N/A |
| Nippert 2002 | Germany | - Retrospective Observational Cohort - Community setting - Primary data, directly collected for this study | Thalidomide | 208 | 38 years | Maternal report | Self-report | Unexposed, general population | N/A |
| Adams 1993 | USA | - Prospective Observational Cohort - Hospital setting - Primary data, directly collected for this study | Isotretinoin | 61 | 5 years | Other: Unclear | Parent; Researcher | Unexposed, general population | N/A |
| Mitchell 1995 | USA | - Prospective Observational Cohort - Hospital setting - Primary data, directly collected for this study | Isotretinoin | 32 | Not Reported | Maternal report | Parent | None | N/A |
| Azizi 2002 | Iran | - Prospective Observational Cohort - Hospital setting - Primary data, directly collected for this study | Methimazole | 53 | 3-11 years | Hospital notes or records | Researcher | Unexposed, general population | N/A – matched on parental education and SES |
| Eisenstein 1992 | Israel | - Retrospective Observational Cohort - Community setting - Primary data, directly collected for this study | Methimazole | 57 | 4-23 years | Hospital notes or records | Researcher | Unexposed, disease matched | N/A |
| McCarroll 1976 | UK | - Retrospective Observational Cohort - Hospital setting - Primary data, directly collected for this study | Carbimazole | 25 | 3-13 years | Hospital notes or records | Researcher | Unexposed, general population | N/A – mothers matched for age, sex, social class and area of origin |
| Chong 1984 | UK | - Retrospective Observational Cohort - Hospital setting - Primary data, directly collected for this study | Warfarin | 41 | 1-5 years | Hospital notes or records | Researcher | Unexposed, general population | N/A |
| Wong 1993 | Hong Kong | - Prospective Observational Cohort - Hospital setting - Primary data, directly collected for this study | Warfarin | 29 | 6m – 11y | Hospital notes or records | Health Professional | None | N/A |
| Hines 1996 | UK | - Retrospective Observational Cohort - Community setting - Primary data, directly collected for this study | DES | 68 | 17-43 years | Hospital notes or records | Researcher | Unexposed, general population | N/A |
| Lish 1991 | USA | - Retrospective Observational Cohort - Community - Primary data, directly collected for this study | DES | 60 | Not Reported | Hospital notes or records | Parent; Self Report | Unexposed, general population | NA |
| Kioumourtzoglou 2018 | USA | - Retrospective Observational Cohort - Community - Primary data, directly collected for this study | DES | 47540 | 27 years | Maternal report | Parent | Unexposed, general population | None Reported |
| Reinisch 1992 | USA | - Retrospective Observational Cohort - Hospital setting - Primary data, directly collected for this study | DES | 20 | 8-21 years | Hospital notes or records | Researcher (blinded) | Unexposed, disease matched | N/A |
| Soyer-Gobillard 2016 | France | - Retrospective Observational Cohort - Community setting - Primary data, directly collected for this study | DES | 1182 | >18 years | Maternal report | Parent; Self Report | Sibling matched - unexposed | N/A |
| Vessey 1983 | UK | - Prospective RCT Cohort - Hospital setting - Secondary use of data collected for another study | DES | 530 | not reported | Hospital notes or records | Health Professional | Unexposed, disease matched | N/A |
| Wilcox 1992 | USA | - Prospective RCT Cohort - Community setting - Primary data, directly collected for this study | DES | 1603 | 17-18 years | Hospital notes or records | Education System | Unexposed, disease matched | Sex |
| Escumalha 2005 | Portugal | - Prospective Case Control Study - Hospital setting - Primary data, directly collected for this study | Misoprostol | 77 | 12 months | Hospital notes or records | Researcher | Unexposed, disease matched | N/A |
| Guedes 2014 | Brazil | - Retrospective Observational Study - Hospital - Primary data, directly collected for this study. | Misoprostol | 18 | 4m – 10y | Hospital notes or records | Health Professional | Unexposed, disease matched | N/A |
