# Supplementary material for: A Scoping Review of Human Teratogens and Their Impact on the Developing Brain: A Contribution From the ConcePTION Project
Source: Birth Defects Res. 2025 Sep 17;117(9):e2497. doi: 10.1002/bdr2.2497 (PMC12442749; doi:10.1002/bdr2.2497)
Supplement: Supplementary file 4 — Supplementary Table 4. Neurodevelopmental outcomes assessed and reported to be significantly altered in ASM cohorts reporting multiple studies. Outcomes may have been measured and/or assessed in one or multiple studies within each cohort. [file BDR2-117-e2497-s001.docx]

Supplementary Table 4: Neurodevelopmental outcomes assessed and reported to be significantly altered in ASM cohorts reporting multiple studies. Outcomes may have been measured and/or assessed in one or multiple studies within each cohort.

| **Cohort** | **Age at Assessment** | **Medication(s)** | **Outcomes Measured** | **Altered Outcomes** |
| --- | --- | --- | --- | --- |
| Australian Pregnancy Register | 6-8 years | CBZ  VPA  TPM | IQ / Intellectual functioning Language development Memory Processing speed  Autistic Spectrum Disorder  Attention  Attention Deficit Hyperactivity Disorders  Behavioural Problems | List Memory   - VPA vs Gen Pop Norms   Non-Verbal Memory   - CBZ vs VPA   Working memory   - VPA vs Gen Pop Norms   Verbal comprehension   - VPA vs Gen Pop Norms   ASD   - Male pre-dominance negated.   Attention   - VPA vs Other ASMs |
| Berlin Cohort | 6-19 years | PB  PHT  VPA  PRM | Behavioural Problems  IQ / Intellectual functioning | Apathy   - PB/PHT vs WWoE   Hyperexcitability   - VPA vs WWoE |
| French National Cohort | 4 years | CBZ  PB  PHT  TPM  VPA | Language development Learning disability diagnosis  Neurodevelopmental Disorders  Autistic Spectrum Disorder Behavioural Problems  Emotional regulation / mood difficulties  Language development | Neurodevelopmental Disorders   - VPA vs WWoE/LTG - CBZ vs WWoE   Pervasive developmental disorders   - VPA vs WWoE/LTG   Mental Retardation   - VPA vs WWoE/LTG   Disorders of Psychological Development   - VPA vs WWoE/LTG - CBZ vs WWoE   Speech Therapy   - VPA vs WWoE/LTG - TPM vs WWoE   Behavioural and Emotional Disorders   - CBZ vs WWoE |
|  |  |  |  |  |
| Danish Health Databases | 6-16 years | CBZ  PB  TPM  VPA | Autistic Spectrum Disorder Attention Deficit Hyperactivity Disorders  Behavioural Problems Emotional regulation / mood difficulties Examination results  Infant global development  Learning difficulty diagnosis Learning disability diagnosis Rates of specialist educational need | Learning Disabilities   - VPA/TPM/PB vs WWE - VPA/PB vs Other AEDs   ASD risk   - VPA vs WWE/WWoE   ADHD risk   - VPA vs WWE/WWoE/LTG - CBZ vs LTG   School performance   - VPA vs WWoE/LTG   Examination results   - CBZ vs WWoE - VPA vs WWoE   Intellectual Disability   - VPA/CBZ vs WWoE   Intellectual Disability with delayed milestones   - VPA/CBZ vs WWoE |
| Dutch EURAP Study | 6-7 years | CBZ  VPA | Attention Deficit Hyperactivity Disorders Autistic Spectrum Disorder; Behavioural Problems Emotional regulation / mood difficulties Learning disability diagnosis  Social Skills  Attention Executive functioning Motor skills (Fine or Gross) IQ / Intellectual functioning Language development Memory Visuo-spatial skills | Psychiatric diagnoses   - VPA vs Other ASMs   Conduct Disorder   - VPA vs Gen Pop Norms   Autistic behaviour   - VPA vs Gen Pop Norms   Social Problems   - VPA vs LTG/LVT   ADHD Symptoms   - VPA vs LVT   FSIQ   - VPA vs LTG:   Verbal IQ   - VPA vs CBZ/LTG/LVT   Attention and Executive Function   - VPA vs CBZ/LTG/LVT   Language   - VPA vs CBZ/LTG/LVT   Memory and Learning   - VPA vs CBZ   Fine Motor, Visuo-Spatial   - VPA vs CBZ/LTG |
| Finnish Pregnancy Register | 6-13 years | CBZ  VPA | Executive functioning IQ / Intellectual functioning Language development Memory  Attention Deficit Hyperactivity Disorders Behavioural Problems  Motor skills (Fine or Gross) Rates of specialist educational need  Social Skills | IQ, Digit Learning, Memory for Faces   - VPA vs CBZ   Digit learning, List learning   - VPA vs WWE |
| Helsinki University Central Hospital | 5-11 years | CBZ  VPA | Attention Motor skills (Fine or Gross) IQ / Intellectual functioning Language development Memory Visuo-spatial skills | Verbal IQ   - VPA vs Other ASMs   Attention, Memory   - VPA vs Other ASMs/WWoE |
| Israeli TIS | 6 months - 7 years | CBZ  TPM  VPA | Attention Deficit Hyperactivity Disorders  Behavioural Problems  Executive functioning  Infant global development  IQ / Intellectual functioning  Motor skills (Fine or Gross) Processing speed  Visuo-spatial skills | Mental/Cognitive development   - CBZ vs WWoE   Motor skills   - VPA/TPM vs WWoE   Behaviour, IQ   - TPM vs WWoE   Sensory processing, Executive function, ADHD symptoms   - VPA vs WWoE |
| Kerala Pregnancy Register | 12 months – 21 years | CBZ  PB  PHT  TPM  VPA | Attention  Infant global development  IQ / Intellectual functioning Language development Memory Motor skills (Fine or Gross) | IQ   - PB vs VPA/CBZ/PHT/WWoE   Language   - VPA vs PB/PHT/CBZ - VPA/PB vs WWoE   Motor Development   - VPA vs Other ASMs/WWE   Mental Development (DQ)   - VPA/PB vs WWE - PB vs WWE |
| Liverpool & Manchester Epilepsy Cohort | 2 months - 6 years | CBZ,  PHT  TPM  VPA, | Autistic Spectrum Disorder  Infant global development  IQ / Intellectual functioning Other Neurodevelopmental Disorders  Rates of specialist educational need | IQ   - VPA vs WWoE/CBZ/LTG   Verbal Abilities (IQ)   - VPA vs LTG - CBZ vs Control   Non-Verbal Abilities (IQ)   - VPA vs CBZ/LTG   Spatial Abilities (IQ)   - VPA vs CBZ/LTG   Educational Intervention   - VPA vs WWoE   ASD Diagnosis   - VPA vs WWoE   Psychomotor Development   - VPA vs WWoE   Risk for NDDs   - VPA vs WWoE |
| Mersey-Manchester Regional Clinics | 6 months -18 years | CBZ  PB  PHT  VPA | Adaptive Behaviour  Behavioural Problems  Infant global development  IQ / Intellectual functioning Language development  Rates of specialist educational need  Social Skills | Additional Educational Needs   - VPA vs WWE   Verbal IQ   - VPA vs Other ASMs/PHT/CBZ/WWE   Daily living, Socialisation, Adaptive behaviour scores   - VPA vs WWE/PHT/CBZ |
| NEAD | 2-6 years | CBZ  PHT  VPA | Adaptive behaviour  Attention  Attention Deficit Hyperactivity Disorders Behavioural Problems Executive functioning  IQ / Intellectual functioning Infant global development Language development  Memory  Motor skills (Fine or Gross) Visuo-spatial skills | Adaptive behaviour, Attention/Atypicality   - VPA vs LTG/PHT   ADHD risk   - VPA/CBZ/LTG vs Gen Pop Estimate   IQ   - VPA/CBZ vs Gen Pop Norms - VPA vs CBZ/PHT/LTG   Cognitive fluency and originality   - VPA vs LTG/CBZ   Verbal and non-verbal cognitive abilities   - VPA vs CBZ/PHT/LTG   DQ   - VPA vs CBZ/PHT/LTG   Attention   - VPA vs Gen Pop Norms   Memory   - VPA vs Gen Pop Norms/CBZ/PHT/LTG   Learning   - VPA/LTG/CBZ vs Gen Pop Norms   Executive Function   - VPA vs CBZ/PHT/LTG |
| North American register | 3-17 years | CBZ  PB  PHT  VPA | Adaptive Behaviour  IQ / Intellectual functioning Language development  Motor skills (Fine or Gross) Social Skills | Adaptive Behaviour   - VPA vs Gen Pop Norms/LTG   Communication, socialisation, motor skills   - VPA vs LTG   IQ   - PB vs WWoE - PB vs PHT |
|  |  |  |  |  |
| Norwegian MoBa Study | 6 months – 8 years | CBZ  TPM  VPA  PB  PHT | Attention Deficit Hyperactivity Disorders Autistic Spectrum Disorder Behavioural Problems  Infant Global Development  Language development  Motor skills (Fine or Gross)  Social Skills | Language   - CBZ/VPA vs WWoE   Personal social, Aggressive symptoms   - CBZ vs WWoE   Motor   - CBZ/VPA/LTG vs WWoE   Autistic Traits   - CBZ vs WWoE |
| Stockholm Cohort | 9 months – 5 years | CBZ  PHT | Social Skills Motor skills (Fine or Gross) Infant global development  Language development  Visuo-Spatial | Locomotor function   - PHT vs WWoE |
| SCAN-AED | 8-18 years | CBZ  PB  TPM  VPA | Autistic Spectrum Disorder  Learning disability diagnosis  Neurodevelopmental Disorders, Attention Deficit/Hyperactivity Disorders  Emotional Regulation/Mood Difficulties | ASD   - CBZ/VPA/TPM vs WWoE   Intellectual Disability   - CBZ/VPA/TPM vs WWoE   Neurodevelopmental Disorders   - CBZ/VPA/TPM vs WWoE   ADHD   - TPM vs WWoE |
| UKEPR | 3 months – 9 years | CBZ  VPA  TPM | Behavioural Problems IQ / Intellectual functioning Infant Global Development Language development Motor skills (Fine or Gross)  Processing speed  Social Skills  Visuo-Spatial | Overall developmental ability, locomotor skills, hand and eye coordination, performance skills, hearing and language.   - VPA vs LVT/WWoE   Gross Motor   - VPA vs LVT/WWoE   Comprehensive Language   - VPA vs LVT/WWoE   Expressive Language   - VPA vs LVT   Overall neurodevelopment   - VPA/CBZ vs WWoE |

CBZ = Carbamazepine, LTG – Lamotrigine, LVT = Levetiracetam, PB = Phenobarbital, PHT = Phenytoin, PRM = Primidone, TPM = Topiramate, VPA = Valproate, WWE = Women with Epilepsy (Unexposed), WWoE = Women without Epilepsy (Unexposed), Gen Pop Norms = General Population Normative Scores/Rates.
