# Supplementary material for: A Scoping Review of Human Teratogens and Their Impact on the Developing Brain: A Contribution From the ConcePTION Project
Source: Birth Defects Res. 2025 Sep 17;117(9):e2497. doi: 10.1002/bdr2.2497 (PMC12442749; doi:10.1002/bdr2.2497)
Supplement: Supplementary file 5 — Supplementary Table 5. Neurodevelopmental outcomes assessed and reported to be significantly altered in ASM cohorts reporting single studies. [file BDR2-117-e2497-s003.docx]

Supplementary Table 5: Neurodevelopmental outcomes assessed and reported to be significantly altered in ASM cohorts reporting single studies.

| **Author** | **Age at Assessment** | **Medication(s)** | **Outcomes Measured** | **Altered Outcomes** |
| --- | --- | --- | --- | --- |
| Arkilo 2015 | 2 years | CBZ  PHT  TPM  VPA | Autistic Spectrum Disorder Motor skills (Fine or Gross) Infant global development  Language development | None |
| Arulmozhi 2006 | 2, 6 & 12 months | CBZ  PHT  VPA | Motor skills (Fine or Gross) Infant global development | Sitting progression   - PHT vs CBZ/VPA |
| Atacan Yasguclukal 2023 | 3m-18 years | CBZ  VPA | Behaviour  Motor skills  Infant global development | Motor skills (sports activity)   - VPA vs Other ASM monotherapy |
| Bluett-Duncan 2023 | 7-37 years | VPA | Attention  Attention Deficit Hyperactivity Disorders  Autistic Spectrum Disorders  Behaviour  Dyspraxia  Emotional Regulation / Mood Difficulties  IQ / Intellectual Functioning  Rates of Special Educational Need  Examination Results  Sensory Difficulties  Other Neurodevelopmental Disorders | IQ / Intellectual Functioning, Academic Functioning, Rates of Special Educational Need, Attention, Mood Difficulties, ASD, ADHD, Dyspraxia, Other Neurodevelopmental Disorders   - FVSD vs VPA^±^/Non-Exposed/Gen Pop Norms |
| Bromley 2019 | 6-27 years | VPA | IQ / Intellectual functioning  Memory  Processing Speed Rates of specialist educational need | FSIQ, Verbal Comprehension, Working Memory, Processing Speed.   - VPA vs Gen Pop Norms   Specialist Educational Need   - VPA: No Comparison |
| Burger 2022 | 10-20 weeks | CBZ  VPA | Motor skills (Fine or Gross) | None |
| Chainirun 2021 | Not Reported | CBZ  PB  PHT  TP  VPA | IQ / Intellectual functioning | Cognitive Impairment   - VPA, PB, PHT (no formal analysis) |
| Charlton 2017 | 6 years | CBZ  VPA | Attention Deficit Hyperactivity Disorders  Autistic Spectrum Disorder Dyspraxia diagnosis | None |
| Dean 2002 | 21 months - 39 years | CBZ  PHT  PB  PRM  VPA | Attention Deficit Hyperactivity Disorders Autistic Spectrum Disorder Motor skills (Fine or Gross) Infant global development  Language development Rates of specialist educational need  Behavioural Problems | Developmental delay, Speech delay, Behaviour disorders   - VPA vs WWoE |
| Dessens 1998 | Not reported | PB  PHT | IQ / Intellectual functioning Visuo-spatial skills Profession | None |
| Dessens 2000 | Not reported | PB  PHT | IQ / Intellectual functioning Memory Rates of specialist educational need | None |
| Forsberg 2011 | 16 years | CBZ  PHT | Examination results | Academic performance   - CBZ vs PHT |
| Gaily 1988 | 5.5 years | PHT | IQ / Intellectual functioning | 1 child with mild intellectual deficiency   - PHT   1 child with severe mental deficiency  CBZ |
| Guveli 2015 | 6-15 years | CBZ  PB  PHT  VPA | Behavioural Problems  IQ / Intellectual functioning | None |
| Hernandez-Diaz 2024 | 8 years | TPM  VPA | Autistic Spectrum Disorder | ASD   - VPA vs Other ASM/WWE |
| Hill 1974 | 9, 12, 18, 21 or 24, and 36 months | PB  PHT  PRM | Infant global development | None |
| Jones 1989 | Varied | CBZ | Infant global development  IQ / Intellectual functioning | Developmental Delay (prospective)   - CBZ: 20%   Developmental Delay (retrospective)   - CBZ: 3 out of 4 children |
| Kasradze 2017 | 36-72 months | CBZ  PB  VPA | IQ / Intellectual functioning | FSIQ, Verbal Comprehension IQ   - VPA vs WWE |
| Kelly 1984 | Varied | CBZ  PB  PHT  VPA | Infant global development | None |
| Kishk 2019 | 5-16 years | CBZ  VPA | IQ / Intellectual functioning | IQ   - VPA vs Non-VPA (WWE + possibly other ASMs) |
| Lacey 2018 | 7 years | CBZ  VPA | Examination results | Educational attainment   - VPA vs WWoE |
| Lajeunie 2001 | 1-72 months | VPA | Infant global development  IQ / Intellectual functioning | DQ/IQ   - VPA: No Comparison |
| Li 2023 | ≤1 year | CBZ  TPM  VPA | Infant global development | Developmental Delay   - VPA vs Other ASM/WWE |
| Meador 2022 | 2 years | CBZ  TPM | Infant global development  Language development | None |
| Millar 1973 | Not reported | PB  PHT  PRM | Infant global development | Mental retardation   - PHT (in one child) |
| MohdYunos 2018 | Not reported | VPA | Attention Deficit Hyperactivity Disorders Autistic Spectrum Disorder Behavioural Problems Infant global development  Language development | Developmental delay, speech delay, ADHD, Aspergers, Disruptive Behaviour   - VPA: No comparison |
| Moore 2000 | 8 months - 16 years | CBZ  PHT  VPA | Attention Deficit Hyperactivity Disorders Autistic Spectrum Disorder; Behavioural Problems IQ / Intellectual functioning Language development Learning difficulty diagnosis Rates of specialist educational need | ASD, Asperges   - VPA (no comparison)   (Majority of findings reported grouped ASMs) |
| Parisi 2003 | 30 months. | CBZ  PB  PRM | Infant global development | Psychomotor Development   - PB: 5/6 children   (No formal analysis of individual AEDs) |
| Putignano 2019 | Up to 1 year | CBZ  VPA | Emotional regulation / mood difficulties | Child Neuropsychiatry Visits   - VPA vs WWoE   (Other ASMs not analysed individually) |
| Rasalam 2005 | 10 years | CBZ  VPA | Autistic Spectrum Disorder | ASD risk   - VPA (no comparison) |
| Reinisch 1995 | Study 1= 23 years Study 2 = 19 years | PB | IQ / Intellectual functioning | IQ, Verbal IQ   - PB vs WWoE |
| Richards 2019 | 4 years | CBZ  VPA | Behavioural Problems Emotional regulation / mood difficulties Motor skills (Fine or Gross) Infant global development  Language development | Emotional/Behavioural problems   - VPA/LTG vs WWoE   Referral for developmental delay/behaviour problems   - CBZ vs WWoE |
| Scolnik 1994 | 18-36 months | CBZ  PHT | Infant global development  IQ / Intellectual functioning Language development | DQ/IQ, Expressive & Receptive Language   - PHT vs WWoE |
| Shankaran 1996 | 12, 24 + 36 months | PB | Motor skills (Fine or Gross) Infant global development  IQ / Intellectual functioning Language development Memory Visuo-spatial skills | None |
| Shankaran 2002 | 18-22 months | PB | Infant global development | None |
| Shapiro 1976 | Not reported | PB  PHT | Motor skills (Fine or Gross) Infant global development  IQ / Intellectual functioning | None |
| Soomro 2024 | ≤4 years | VPA | Attention Deficit Hyperactivity Disorders | ADHD   - VPA vs Other ASM/WWoE |
| Thorp 2003 | 7 years | PB | Behavioural Problems IQ / Intellectual functioning | None |
| vanderPol 1991 | 7-13 years | CBZ  PB | Behavioural Problems  Attention IQ / Intellectual functioning | Arithmetic, Spelling, Concentration   - PB vs WWoE   Attention   - PB/CBZ vs WWoE |
| Videman 2016 | 7 months | CBZ  TPM  VPA | Motor skills (Fine or Gross) Infant global development  Language development  Social Skills | Hearing, Speech   - CBZ/VPA vs WWoE   Personal-Social   - CBZ vs WWoE   Overall development   - VPA vs WWoE |
| Wiggs 2020 | 2-10 years | CBZ,  VPA | Attention Deficit Hyperactivity Disorders  Autistic Spectrum Disorder | ASD   - VPA vs WWoE   ADHD   - VPA vs WWoE |
| Yigin 2021 | NR | CBZ  VPA | Behavioural Problems | Behavioural Defects   - VPA vs WWoE |

CBZ = Carbamazepine, LTG – Lamotrigine, LVT = Levetiracetam, PB = Phenobarbital, PHT = Phenytoin, PRM = Primidone, TPM = Topiramate, VPA = Valproate, WWE = Women with Epilepsy (Unexposed), WWoE = Women without Epilepsy (Unexposed), FVSD = Fetal Valproate Spectrum Disorder, Gen Pop Norms = General Population Normative Scores/Rates. ± Participants were exposed to VPA but had not received a diagnosis of FVSD.
