# Supplementary material for: A Scoping Review of Human Teratogens and Their Impact on the Developing Brain: A Contribution From the ConcePTION Project
Source: Birth Defects Res. 2025 Sep 17;117(9):e2497. doi: 10.1002/bdr2.2497 (PMC12442749; doi:10.1002/bdr2.2497)
Supplement: Supplementary file 6 — Supplementary Table 6. Neurodevelopmental outcomes assessed and reported to be significantly altered in cohorts reporting exposure to other medications. [file BDR2-117-e2497-s006.docx]

Supplementary Table 6: Neurodevelopmental outcomes assessed and reported to be significantly altered in cohorts reporting exposure to other medications.

| **Author** | **Age at Assessment** | **Medication(s)** | **Outcomes Measured** | **Altered Outcomes** |
| --- | --- | --- | --- | --- |
| Imai 2014 | 47-51 years | Thalidomide | Autistic Spectrum Disorder Emotional regulation / mood difficulties IQ / Intellectual functioning  Social Skills  Memory  Processing Speed | General mental health problems, Somatic Symptoms, Anxiety and Insomnia, Social Dysfunction, Working Memory, Processing Speed   - Thalidomide vs Gen Pop Norms |
| Imai 2020 | 54 years | Thalidomide | Emotional regulation / mood difficulties | High levels of mental health problems   - Thalidomide (no comparison) |
| Kanno 1987 | 7-22 years | Thalidomide | IQ / Intellectual functioning | IQ Impairment   - Thalidomide (no comparison) |
| McFie 1973 | 7-10 years | Thalidomide | Adaptive Behaviour IQ / Intellectual functioning | Intelligence, Adaptive Skills, Spelling and Reading   - Thalidomide (no comparison) |
| Mongeau 1966 | 32-41 months | Thalidomide | Infant global development  IQ / Intellectual functioning  Language development | Language Development   - Thalidomide (no comparison) |
| Nippert 2002 | 38 years | Thalidomide | Emotional regulation / mood difficulties | None |
| Adams 1993 | 5 years | Isotretinoin | Attention Behaviour Problems Executive functioning Motor skills (Fine or Gross) IQ / Intellectual functioning Language development Visuo-spatial skills | General Mental Ability, Attention, Visual-Motor Integration, Visual Perceptual Abilities, Organisational Abilities.   - Isotretinoin vs Non Exposed |
| Mitchell 1995 | Not Reported | Isotretinoin | Infant global development | 1 child reported to have developmental delay   - Isotretinoin (no comparison) |
| Azizi 2002 | 3-11 years | Methimazole | IQ / Intellectual functioning | None |
| Eisenstein 1992 | 4-23 years | Methimazole | IQ / Intellectual functioning | None |
| McCarroll 1976 | 3-13 years | Carbimazole | Behaviour Problems IQ / Intellectual functioning | None |
| Chong 1984 | 1-5 years | Warfarin | Infant global development | None |
| Wong 1993 | 6 months - 11 years | Warfarin | Infant global development  IQ / Intellectual functioning | IQ   - Warfarin: 2/18 children   Delayed Development   - Warfarin: 1/18 |
| Hines 1996 | 17-43 years | DES | Language development Memory Processing speed Visuo-spatial skills | None |
| Kioumourtzoglou 2018 | NR | DES | Attention Deficit Hyperactivity Disorders | ADHD (in 3rd generation)   - DES VS Non Exposed |
| Lish 1991 | 27 years | DES | Behaviour Problems | None |
| Reinisch 1992 | 8-21 years | DES | IQ / Intellectual functioning Visuo-spatial skills | Spatial Ability   - DES vs Control |
| Soyer-Gobillard 2016 | >18 years | DES | Behavioural Problems Emotional regulation / mood difficulties | Behavioural Disorders, Eating Disorders, Schizophrenia, Depression, Suicide attempts   - DES vs Non Exposed/Post-DES |
| Vessey 1983 | not reported | DES | Behavioural Problems Emotional regulation / mood difficulties Learning disability diagnosis | Psychiatric Disorders   - DES vs Non Exposed |
| Wilcox 1992 | 17-18 years | DES | Examination results | Social Science   - Non Exposed vs DES |
| Escumalha 2005 | 12 months | Misoprostol | Infant global development | Global Developmental Delay   - Misoprostol vs Non Exposed |
| Guedes 2014 | 4 months – 10 years | Misoprostol | Language Development | None |

DES = Diethylstilbestrol, Gen Pop Norms = General Population Normative Scores/Rates.
