# Supplementary material for: A Scoping Review of Human Teratogens and Their Impact on the Developing Brain: A Contribution From the ConcePTION Project
Source: Birth Defects Res. 2025 Sep 17;117(9):e2497. doi: 10.1002/bdr2.2497 (PMC12442749; doi:10.1002/bdr2.2497)
Supplement: Supplementary file 7 — Supplementary Table 7. Timing of approval and publications for each included medication. [file BDR2-117-e2497-s007.docx]

Supplementary Table 7: Timing of approval and publications for each included medication.

| Medication | Approved | Case Series/Reports | | | Empirical Cohorts | | | Time between first adverse case report and first Empirical Cohort^ƚ^ |
| --- | --- | --- | --- | --- | --- | --- | --- | --- |
|  |  | Total | Range | Time to first study | Total | Range | Time to first study^‑ƚ^ |  |
| **Anti-Seizure** | | | | | | | |  |
| Carbamazepine | 1965^a^ | 15 | 1980-2020 | 15 years | 39 | 1984-2023 | 26 years | 11 years |
| Phenytoin | 1953^b^ | 32 | 1972-2019 | 19 years | 24 | 1973-2022 | 23 years | 4 years |
| Fosphenytoin | 1996^c^ | 0 | N/A | N/A | 0 | N/A | N/A | N/A |
| Primidone | 1954^d^ | 9 | 1972-1995 | 18 years | 5 | 1973-2003 | 45 years | 24 years |
| Topiramate | 1996^e^ | 0 | N/A | N/A | 13 | 2010-2024 | 14 years | N/A |
| Valproate | 1967^f^ | 40 | 1981-2021 | 14 years | 38 | 1984-2024 | 29 years | 15 years |
| Phenobarbital | 1912^g^ | 20 | 1972-2016 | 60 years | 22 | 1973-2023 | 64 years | 3 years |
| **Anticoagulant** | | | | | | | |  |
| Phenindione | 1952^h^ | 0 | N/A | N/A | 0 | N/A | N/A | N/A |
| Warfarin | 1954^i^ | 14 | 1975-2013 | 21 years | 2 | 1984-1993 | 30 years | 8 years |
| Acenocoumarol | 1957^j^ | 2 | 2002-2013 | 45 years | 0 | N/A | N/A | N/A |
| **Antithyroid** | | | | | | | |  |
| Carbimazole | 1953^k^ | 3 | 2005-2012 | 52 years | 1 | 1976 | 23 years | N/A |
| Methimazole | 1949^l^ | 11 | 1975-2020 | 26 years | 2 | 1992-2002 | 43 years | 5 years |
| **Immunosuppressive** | | | | | | | |  |
| Mycophenolate | 1995^m^ | 5 | 2008-2021 | 13 years | 0 | N/A | N/A | N/A |
| Methotrexate and Aminopterin | 1953^n^ | 7 | 1968-2005 | 15 years | 0 | N/A | N/A | N/A |
| **Oral Retinoids** | | | | | | | |  |
| Acitretin | 1996^o^ | 1 | 2004 | 8 years | 0 | N/A | N/A | N/A |
| Alitretinoin | 1999^p^ | 0 | N/A | N/A | 0 | N/A | N/A | N/A |
| Bexarotene | 1999^q^ | 0 | N/A | N/A | 0 | N/A | N/A | N/A |
| Isotretinoin | 1982^r^ | 8 | 1985-2010 | 3 years | 2 | 1993-1995 | 11 years | 8 years |
| Tretinoin | 1971^s^ | 0 | N/A | N/A | 0 | N/A | N/A | N/A |
| **Other Medications** | | | | | | | |  |
| Thalidomide | 1957^t^ | 2 | 1994-2013 | 37 years | 6 | 1966-2020 | 57 years | 8 years |
| Lenalidomide | 2005^u^ | 0 | N/A | N/A | 0 | N/A | N/A | N/A |
| Misoprostol | 1988^v^ | 4 | 1993-2013 | 5 years | 2 | 2005-2014 | 17 years | 12 years |
| Diethylstilbesterol | 1938^w^ | 0 | N/A | N/A | 7 | 1983-2018 | 45 years | N/A |

^ƚ^ First study that includes comparison group and formal analysis of data.

^a^ <https://www.ncbi.nlm.nih.gov/pmc/articles/PMC3743329/>

^b^ <https://www.accessdata.fda.gov/drugsatfda_docs/label/2021/084349s087lbl.pdf>

^c^ <https://www.drugs.com/history/sesquient.html>

^d^ <https://go.drugbank.com/drugs/DB00794>

^e^ <https://go.drugbank.com/drugs/DB00273>

^f^ <https://doi.org/10.1016/S1474-4422(15)00398-1>

^g^ <https://www.ncbi.nlm.nih.gov/pmc/articles/PMC2424120/>

^h^ <https://drugs.ncats.io/d>

^I^ <https://www.hematology.org/about/history/50-years/milestones-anticoagulant-drugs>

^j^ <https://drugs.ncats.io/drug/I6WP63U32H>

^k^ <https://doi.org/10.1210/jcem-16-3-391>

^l^ <https://www.ncbi.nlm.nih.gov/books/NBK548406/>

^m^ <https://www.drugs.com/history/cellcept.html#:~:text=CellCept%20FDA%20Approval%20History&text=CellCept %20has%20been%20approved%20for,transplant%20on%20July%2028%2C%202000>

^n^ <https://go.drugbank.com/salts/DBSALT000115>

^o^ <https://www.drugs.com/availability/generic-soriatane.html>

^p^ <https://www.accessdata.fda.gov/drugsatfda_docs/nda/99/20886.cfm#:~:text=Approval%20Date%3A%202%2F2 %2F1999>

^q^ <https://www.drugs.com/availability/generic-targretin.html>

^r^ <https://go.drugbank.com/drugs/DB00982>

^s^ <https://www.accessdata.fda.gov/scripts/cder/daf/index.cfm?event=overview.process&ApplNo=016921>

^t^ <https://www.ncbi.nlm.nih.gov/pmc/articles/PMC4737249/>

^u^ <https://www.drugs.com/history/revlimid.html>

^v^ <https://go.drugbank.com/drugs/DB00929>

^W^ <https://www.uptodate.com/contents/outcome-and-follow-up-of-diethylstilbestrol-des-exposed-individuals>
